# Sleep starts: Phenomenology and associations with sleep, psychological, and demographic characteristics

**DOI:** 10.1371/journal.pmen.0000737

**Published:** 2026-11-11

**Authors:** Tanja Grünberger, Christopher Höhn, Manuel Schabus, Anton-Rupert Laireiter

**Affiliations:** Department of Psychology, Paris-Lodron-University Salzburg, Salzburg, Austria; University of Tübingen fMEG Center, GERMANY

## Abstract

Sleep starts (SS) are transient phenomena during sleep onset. Although traditionally characterized by sudden muscle jerks, they may also involve sensory, cognitive, emotional, and autonomic experiences. Despite their high prevalence, empirical data on phenomenology and associated characteristics remain limited. This online survey examined SS phenomenology and exploratory associations with demographic, sleep-related, and psychological variables in 194 adults. Participants with self-reported sleep-related movement disorders or relevant medical conditions were excluded. Individuals reporting abrupt awakenings ≥1/week and ≥1 additional symptom formed the SS group (SS frequency ≥1, n = 96); all others the comparison group (SS frequency <1, n = 98). Because SS were operationalized using self-reported symptoms, they could not be distinguished from related sleep-onset phenomena (e.g., exploding head syndrome, hypnagogic hallucinations). Standardized questionnaires (PSQI, ISI, ESS, MZS, HADS) assessed sleep quality, insomnia severity, daytime sleepiness, dysfunctional sleep-related beliefs, and anxiety/depression symptoms. Participants described diverse experiences including motor jerks, falling sensations, dream-like experiences, somatosensory symptoms, autonomic arousal, and emotional reactions, accompanied by alarm, anxiety, and confusion. Compared with controls, the SS group reported poorer sleep quality, greater insomnia severity, stronger dysfunctional sleep-related beliefs, and higher anxiety/depressive symptoms (all p < .05). Exploratory regression analyses indicated that poorer sleep quality, stronger dysfunctional sleep-related beliefs, and higher anxiety symptoms were associated with higher SS frequency, whereas depressive symptoms were not. The final model explained 26.9% of variance (R^2^ = .269, adjusted R^2^ = .245). More frequent SS were retrospectively reported during emotional stress, whereas sleep deprivation was perceived as less influential. Although many described SS as manageable, a substantial subgroup reported distress and sleep disruption. This suggests that SS represent heterogeneous sleep-onset experiences associated with subjective sleep disturbance and cognitive-emotional arousal. Although causality cannot be inferred from cross-sectional self-report data, the results highlight potentially relevant links between SS and sleep-related cognitive-emotional processes.

## 1 Introduction

As wakefulness transitions into sleep, many individuals occasionally experience sudden muscle jerks, sensations of falling, or brief autonomic arousal that leads to immediate awakening. These phenomena are commonly referred to as sleep starts (SS) or hypnic jerks and are considered a frequent and generally benign feature of sleep onset. Prevalence estimates suggest that approximately 60–70% of individuals experience SS at least sporadically, with around 10% reporting daily occurrences, although many events may not be recalled upon full awakening [1,2].

Defining sleep starts is challenging due to heterogeneous terminology, varying manifestations, and substantial overlap with related phenomena. The overarching term for sudden, involuntary muscle jerks is myoclonus. One subtype, hypnoclonus, refers to jerks occurring during the transition from wakefulness to sleep. These events are typically described as isolated, usually asymmetric muscle contractions affecting individual limbs or the entire body and may occur repeatedly. They are frequently accompanied by abrupt awakening, a sensation of falling, and autonomic activation such as palpitations [1,3].

The terminology surrounding sleep-onset jerks remains heterogeneous. The umbrella term myoclonus includes various involuntary muscle contractions, with hypnic (sleep-onset) myoclonus representing a subtype occurring during the transition from wakefulness to sleep. These events are typically characterized by brief, isolated, and sometimes asymmetric muscle contractions that may involve a single limb or the entire body and can be accompanied by a sensation of falling or sudden awakening with autonomic activation [1,3].

In the literature, the terms hypnic jerks and sleep starts are often used interchangeably, although some authors distinguish between motor, sensory, and mixed sensorimotor-emotional manifestations [3,4]. In the present study, the term sleep starts (SS) is used as an umbrella construct encompassing motor, sensory, and affectively modulated experiences occurring at sleep onset.

SS may include sensory and perceptual phenomena such as tingling sensations, auditory distortions, flashes of light, or hypnagogic dream fragments [1,3]. In more intense cases, abrupt awakenings may be accompanied by fear, panic, or sensations resembling electrical shocks. Although generally considered benign, more pronounced SS have been associated with sleep-onset insomnia and anticipatory anxiety about sleep [1,5,6]. These more severe manifestations have been described as intensified sleep starts and appear to be associated with heightened anxiety and a general predisposition toward anxiety [1,6].

Differential diagnosis includes restless legs syndrome, periodic limb movement disorder, epilepsy, and other neurological or sleep-related movement disorders [2,3]. Particularly challenging is the distinction from exploding head syndrome (EHS), as both conditions may include perceptions such as loud explosive noises and flashes of light [1,3,7]. Although EHS is classified as a distinct parasomnia, the considerable phenomenological overlap has also led some authors to describe it as a purely sensory form of sleep starts (cf. [1,7]. Given this overlap, SS in the present study are conceptualized as a broader phenomenological spectrum that may include features overlapping with EHS rather than as strictly separable entities, as fine-grained diagnostic differentiation is not feasible within the constraints of an anonymous online self-report design.

Hypnagogic hallucinations are typically more complex perceptual experiences, often visual, and are more strongly associated with REM intrusion phenomena, although overlap with sleep-onset imagery may occur [1].

In most cases, no underlying pathological cause for SS can be identified. It has frequently been suggested that factors such as caffeine and other stimulants, intense physical activity, sleep deprivation, and emotional stress may increase the frequency or severity of SS [2,8,9]. However, to the best of our knowledge, these assumptions have not yet been systematically substantiated. A review chapter on sleep-disrupting movement disorders similarly notes that treatment recommendations are largely based on expert consensus because randomized controlled trials remain scarce [9], p. 346). Nevertheless, there is some evidence that selective serotonin reuptake inhibitors may induce SS [5,10,11].

Polysomnographic single-case studies indicate that SS occur during quiet wakefulness and stage N1 sleep, but not during N3 or REM sleep [9]. Electromyography (EMG) typically demonstrates brief, high-amplitude muscle potentials, whereas electroencephalography (EEG) commonly shows drowsiness-related activity or N1 sleep patterns [1]. In some cases, a “negative-vertex sharp wave” has also been reported, although the corresponding EEG derivation was not specified in the original reports [1,9]. SS are frequently accompanied by sympathetic activation, including tachycardia and tachypnea, as well as transient lightening of sleep [9].

The neurophysiological mechanisms underlying SS remain incompletely understood. One hypothesis proposes that instability during the wake–sleep transition triggers sudden descending volleys originating in the brainstem reticular formation, resulting in the characteristic motor jerk [1]. The phenomenological similarity between SS and the startle response has further led to the suggestion that abnormal sensory processing may constitute the primary event, whereas motor manifestations arise secondarily through activation of reticulospinal pathways. In this framework, externally triggered startle responses during wakefulness and spontaneous startle-like events during sleep onset may share common neurophysiological mechanisms. An alternative interpretation is that some SS represent motor responses to hypnagogic imagery, such as dreams of falling or sudden loss of balance [1].

Although SS are generally considered benign, more pronounced manifestations may nevertheless cause considerable distress and lead affected individuals to seek treatment or relief. Current recommendations primarily consist of reassurance regarding the harmless nature of the phenomenon, optimization of sleep hygiene, and avoidance of presumed triggering factors. In severe cases, low-dose benzodiazepines, particularly clonazepam, have been described as a treatment option [1–3,5,12,13]. However, these recommendations are based on limited empirical evidence.

Despite the high prevalence of SS, research remains scarce and consists predominantly of individual polysomnographic case reports and small clinical observations [2,5]. While such studies provide valuable information regarding physiological correlates, they offer only limited insight into the subjective experience of SS. Thoughts, emotions, bodily sensations, perceived triggers, distress, and coping strategies can only be investigated through self-report and remain largely understudied.

Accordingly, the present study pursued two objectives. First, it aimed to provide a detailed phenomenological description of SS from the perspective of affected individuals using both quantitative and qualitative data. Second, it examined whether demographic, sleep-related, and psychological characteristics are associated with SS frequency. Given the limited empirical literature, all analyses addressing these associations were considered exploratory in nature.

The study addressed the following research questions:

How are SS subjectively experienced and described, particularly with regard to motor responses, cognition, emotions, bodily sensations, perceptions, and behavioral consequences?
2. Which demographic, sleep-related, and psychological characteristics are associated with SS frequency?

The present investigation focused on age, gender, subjective sleep quality, sleep efficiency, sleep-onset latency, total sleep time, insomnia severity, daytime sleepiness, dysfunctional sleep-related beliefs, anxiety, depression, private and work-related concerns, and pre-existing physical or mental health conditions.

## 2 Materials and methods

### 2.1 Ethics statement

At the time of data collection, the study was considered exempt from formal ethics review because it involved anonymous online self-report data only. This exemption was confirmed retrospectively by the Ethics Committee of the University of Salzburg (reference number GZ 49/2025: “The study qualified for an ethical exemption by the standards of the Ethics Committee of the University of Salzburg.”). Participants received detailed information regarding study aims, procedures, and data handling before providing informed consent. No identifying information was collected.

The study was conducted and reported in accordance with STROBE guidelines.

### 2.2 Study design and participants

This cross-sectional online survey investigated the subjective phenomenology of sleep starts (SS) and their associations with demographic, sleep-related, and psychological characteristics. Data were collected between June 2020 and January 2021 using the open-source online survey platform LimeSurvey (https://www.limesurvey.org).

Participants were recruited in Austria and Germany via mailing lists, flyers, social media platforms, and direct contact with general practitioners and rehabilitation clinics. Participation was voluntary, unpaid, and not restricted to invited individuals or specific patient groups. Electronic informed consent was obtained before survey access. All data were collected and processed anonymously.

The target population comprised adults from the general population, regardless of SS experience. This recruitment strategy enabled comparisons between participants with and without SS without restrictive preselection criteria in a field with limited prior evidence. Convenience sampling was employed, with self-selection into the study and no random sampling.

Predefined exclusion criteria included self-reported diagnoses that may represent differential diagnoses or major confounders of SS, namely restless legs syndrome (RLS), periodic limb movement disorder (PLMD), sleep apnea, epilepsy, Parkinson’s disease, and other neurological or cardiological disorders.

Given the substantial phenomenological overlap between SS, exploding head syndrome (EHS), and hypnagogic hallucinations, reliable differentiation based on anonymous self-report was considered impossible. Consequently, these phenomena were not used as exclusion criteria, as this would have implied a level of diagnostic precision not achievable within the methodological constraints of the study.

Participants were assigned to the SS group (FoSS ≥ 1) if they reported sudden awakenings during sleep onset without an identifiable external trigger and endorsed one or more additional SS-related symptoms (motor, sensory, or perceptual) with a frequency of at least “rarely”. During data inspection, responses indicated substantial heterogeneity in reported SS frequency, including very rare events (e.g., approximately one episode per year) that were associated with minimal phenomenological detail and increased variability in descriptive analyses. To reduce the influence of such extreme low-frequency cases on the characterization of SS phenomenology and related statistical estimates, a frequency criterion of at least one SS episode per week (FoSS ≥ 1) was introduced post hoc as a data-driven refinement. This decision was motivated by observed response distributions and aimed at improving the stability and interpretability of group-level analyses rather than altering the symptom-based definition of SS. To assess the robustness of this post hoc determined threshold, sensitivity analyses were conducted.

### 2.3 Sample size and power analysis

An a priori power analysis assumed medium effect sizes (*Cohen’s d* = 0.50; *r* = 0.30). Considering multiple testing and conservative significance thresholds approximating Benjamini–Hochberg correction (*q* = .05), required sample sizes ranged from *N* = 134 to *N* = 200 for group comparisons and from *N* = 82 to *N* = 122 for correlation analyses, depending on the alpha level. The final sample (*N* = 194) was therefore considered sufficient for the planned analyses.

### 2.4 Participant flow

A total of 314 individuals accessed the survey. 58 participants discontinued the survey before any data were stored and were excluded from analyses, leaving 256 eligible cases. Of these, 55 discontinued participation before completing the questionnaire and were excluded since meaningful evaluation was not possible. An additional seven participants were excluded based on predefined medical criteria (RLS, PLMD, sleep apnea, Parkinson’s disease), resulting in a final sample of 194 participants (96 with SS at least weekly and 98 with no or very rare SS). Table 1 summarizes participant recruitment and exclusion procedures.

**Table 1:** Participant flow.

*Stage*	*N*
Website visits	314
Excluded (no data storage)	–58
Eligible cases	256
Survey discontinuation	–55
Completed datasets	201
Excluded (medical criteria)	–7
**Final sample**	**194**

Because all questionnaire items were mandatory, missing data occurred only through survey discontinuation. No duplicate entries were identified based on manual inspection of demographic characteristics and response patterns. The overall sample had a mean age of 32.79 years (*SD* = 13.34), and 148 participants identified as female.

### 2.5 Measures

The survey combined standardized questionnaires with study-specific items developed to assess SS phenomenology.

The first section assessed demographic and background characteristics, including age, gender, employment status, sleeping arrangements, physical health conditions, and mental health history. Current occupational and personal stressors were assessed using two open-ended questions.

Participants reporting SS completed an additional SS-specific module assessing symptom phenomenology, frequency, perceived consequences, influencing factors, and coping strategies (Table 2). Frequency was assessed as both the average number of nights with SS per week and the average number of SS per night. Additional items assessed motor, sensory, emotional, cognitive, and distress-related experiences associated with SS.

**Table 2:** Overview of self-developed items assessing sleep start phenomenology: Questionnaire domains, example items, and response formats.

*Domain*	*Example item*	*Response format*
Frequency of SS	How often does startling awake during sleep onset occur for you on average?	Open numeric response(per night / nights per week)
SS-related symptoms	How often do you experience any of the following when startling awake during sleep onset?	Multiple-choice + frequency scale
Emotional reactions	How do you typically feel immediately after startling awake?	Multiple-choice + frequency scale
Distress	Do you feel distress from- the startling awake itself,- the fear of negative consequences or- actual negative consequences of startling awake?	Likert scale
Perceived consequences	Have any long-term consequences occurred as a result of startling awake?	Multiple-choice + frequency scale
Triggering/modulating factors	Does startling awake become more or less frequent under certain conditions?	Multiple-choice + frequency scale
Coping strategies	Have you tried methods to reduce startling awake?	Multiple-choice + frequency scale

Because no validated instrument for SS phenomenology exists, all SS-specific items were developed based on previous literature [1–3]. Items were reviewed internally for comprehensibility before deployment. The survey was administered in German; English translations are provided for reporting purposes. Response formats included Likert scales, categorical response options, open-ended responses, and numerical frequency estimates. Participants were instructed to report their typical experiences. The complete questionnaire is available in S1 Appendix.

Standardized measures included the Pittsburgh Sleep Quality Index (PSQI; [14,15]) to assess global sleep quality (possible range 0–21), with higher scores indicating poorer sleep quality. In addition to the PSQI total score, subjective sleep quality (SSQ), sleep-onset latency (SOL), sleep efficiency, and total sleep time (TST) were derived from PSQI items.

Daytime sleepiness was assessed using the Epworth Sleepiness Scale (ESS; [16,17]; possible range 0–24). Insomnia severity was measured using the Insomnia Severity Index (ISI; [18,19]; possible range 0–28). Dysfunctional sleep-related cognitions were assessed using the German version of the Dysfunctional Beliefs and Attitudes About Sleep Scale (DBAS-16), the Meinungen-zum-Schlaf-Fragebogen (MZS; [20,21]). The MZS consists of 16 items rated from 0 to 10, with higher scores indicating stronger dysfunctional beliefs about sleep.

Subjective importance of sleep was assessed using a single item (“How important is your sleep to you?”) rated on a five-point scale ranging from 1 (“It is not important to me; I do not think about my sleep”) to 5 (“I organize my life around not jeopardizing my sleep”).

Symptoms of anxiety and depression were assessed using the German version of the Hospital Anxiety and Depression Scale (HADS; [22,23]). Possible scores range from 0 to 42 for the total scale and from 0 to 21 for each subscale, with higher scores indicating greater symptom severity.

### 2.6 Statistical analyses

SS phenomenology was investigated using both quantitative and qualitative methods.

Quantitative analyses of SS characteristics were restricted to participants meeting SS-group criteria (FoSS ≥ 1 and one or more additional SS-related symptoms at least “rarely”). Descriptive statistics were calculated for phenomenological items.

Qualitative analyses included all available open-ended responses and were conducted using inductive thematic analysis. Categories were derived directly from the data, and category frequencies were recorded. Responses containing multiple themes could be assigned to more than one category.

Group differences between participants reporting at least weekly SS and those reporting less frequent or no SS (comparison group) were examined using Mann–Whitney U tests and χ^2^ tests. Associations between FoSS and continuous variables within the SS group (FoSS ≥ 1) were assessed using Spearman rank correlations. Effect sizes were reported as *r* for Mann–Whitney U tests and *Cramer’s V* for χ^2^ tests and interpreted according to Cohen’s recommendations [24].

To examine variables jointly associated with SS frequency, blockwise multiple regression analyses were conducted using log-transformed FoSS scores [ln(FoSS + 1)] as the dependent variable. Age and gender were included as covariates in the first block, followed by sleep-related variables (PSQI and MZS) in the second and psychological variables (HADS anxiety and depression) in the third block. Given the limited empirical evidence on SS, the present study was exploratory. Analyses were selected to provide complementary perspectives:

Group comparisons were conducted to examine differences between the SS group (FoSS ≥ 1) and the comparison group (FoSS < 1). Correlation analyses assessed bivariate associations between SS frequency and continuous variables. Hierarchical regression analyses were performed to identify variables that explain variance in SS frequency. These approaches address distinct levels of analysis (group differences, associations, and multivariate models) and were considered complementary and of equal exploratory status. As sensitivity analyses, group comparisons and correlational analyses were repeated using the 25^th^ and 75^th^ percentiles as alternative cut-offs to assess the robustness of the post hoc-determined threshold used for group classification.

False discovery rate (FDR) correction was applied separately to group comparisons and correlation analyses using the Benjamini–Hochberg procedure [25,26] (*q* = .05). Regression analyses were not subjected to FDR correction because they represented multivariate models rather than families of independent tests.

Assumptions regarding normality, outliers, and multicollinearity were examined prior to analysis. Outliers were retained because they were considered plausible observations and sensitivity analyses indicated no meaningful impact on the results.

All analyses were conducted using SPSS version 32 [27].

## 3 Results

### 3.1 Phenomenology of sleep starts

Multiple symptoms could be reported concurrently; therefore, item-level frequencies are not mutually exclusive. Table 3 presents descriptive statistics of the primary phenomenological categories, distress related to SS and perceived changes in SOL. A graphical representation is provided in Figs 1, Fig 2, Fig 3, Fig 4, Fig 5–Fig 6. Additional categories and detailed item-level distributions are available in S1 Appendix.

**Table 3:** Sleep start phenomenology and associated experiences.

*Characteristic*	*M (95% CI)*	*SD*	*Md*	*n (%) ≥ 2*
**Accompanying sensations / symptoms (Fig 1)** *Response format: never (1), rarely (2), occasionally (3), often (4), very often (5)*				
Muscle jerks or twitches	2.97 (2.72–3.21)	1.21	3.00	84 (87.50%)
Dream during sleep onset	2.49 (2.24–2.74)	1.22	2.00	71 (73.96%)
Falling or fainting sensations	2.33 (2.09–2.58)	1.22	2.00	63 (65.62%)
Palpitations or rapid breathing	2.23 (1.98–2.48)	1.24	2.00	60 (62.50%)
Tingling, numbness, or itching	2.21 (1.97–2.44)	1.16	2.00	61 (63.54%)
Electric-shock-like sensations	1.91 (1.66–2.15)	1.20	1.00	43 (44.79%)
Visual phenomena	1.88 (1.65–2.10)	1.11	1.00	46 (47.92%)
Abrupt startle reactions	1.74 (1.52–1.96)	1.07	1.00	38 (39.58%)
Auditory phenomena	1.38 (1.21–1.54)	0.82	1.00	21 (21.88%)
**Accompanying emotions (Fig 2)** *Response format: never (1), rarely (2), occasionally (3), often (4), very often (5)*				
alarmed/alert	3.00 (2.72–3.28)	1.37	3.00	76 (79.17%)
confused/disoriented	2.94 (2.66–3.21)	1.36	3.00	76 (79.17%)
anxious/worried	2.85 (2.55–3.15)	1.48	3.00	71 (73.96%)
frightened/panic/threat perception	2.80 (2.51–3.09)	1.43	3.00	69 (71.87%)
wide awake/clear/fully alert	2.54 (2.30–2.78)	1.20	2.00	75 (78.12%)
neutral	2.47 (2.22–2.72)	1.23	2.00	73 (76.04%)
desperate/sad/depressed	2.14 (1.87–2.40)	1.30	2.00	52 (54.17%)
**Short-term consequences (Fig 3)** *Response format: never (1), rarely (2), occasionally (3), often (4), very often (5)*				
Rapid return to sleep	3.34 (3.12–3.57)	1.09	4.00	92 (95.83%)
Trying to force sleep onset	2.75 (2.48–3.02)	1.33	3.00	73 (76.04%)
Fully awake, unable to resume sleep	2.36 (2.14–2.59)	1.10	2.00	73 (76.04%)
Fear of recurrence	1.84 (1.59–2.10)	1.24	1.00	38 (39.58%)
Concern about next-day consequences	1.81 (1.59–2.04)	1.11	1.00	42 (43.75%)
Concern about health causes	1.74 (1.50–1.98)	1.18	1.00	34 (35.42%)
Concern about health consequences	1.52 (1.30–1.74)	1.08	1.00	24 (25.00%)
**Accompanying cognitions (Fig 4)** *Response format: never (1), rarely (2), occasionally (3), often (4), very often (5)*				
Strong pressure to fall asleep	3.15 (2.88–3.41)	1.32	3.00	80 (83.33%)
Private concerns	2.78 (2.52–3.04)	1.28	3.00	77 (80.21%)
Everyday thoughts	2.63 (2.37–2.88)	1.27	3.00	72 (75.00%)
Work-related concerns	2.27 (2.04–2.50)	1.13	2.00	66 (67.75%)
Expectation of sleep onset	1.96 (1.70–2.22)	1.27	1.00	42 (43.75%)
“Must not fall asleep” thoughts	1.39 (1.22–1.55)	0.81	1.00	23 (23.96%)
**Distress related to… (Fig 5)** *Response format: Not at all (1), slightly (2), moderately (3), strongly (4), very strongly (5)*				
…the SSs themselves	2.18 (1.98–2.38)	1.00	2.00	72 (75.00%)
… actual consequences of SSs	1.82 (1.63–2.02)	0.97	2.00	52 (54.17%)
…concerns about anticipated negative consequences of SSs	1.68 (1.50–1.86)	0.89	1.00	46 (47.92%)
**Perceived changes in sleep onset latency (SOL) due to SS (Fig 6)**				
SOL extension	43.36 (30.63–56.09)	42.86	30.00	49 (51.04%)
SOL shortening	21.39 (9.74–33.04)	15.16	20.00	9 (9.38%)
SOL not affected				39 (40.63%)

**Note.**

n (%) ≥ 2: number and percentage of participants in the SS group reporting the respective answer optionat least “rarely” (response score ≥ 2).

**Fig 1:**
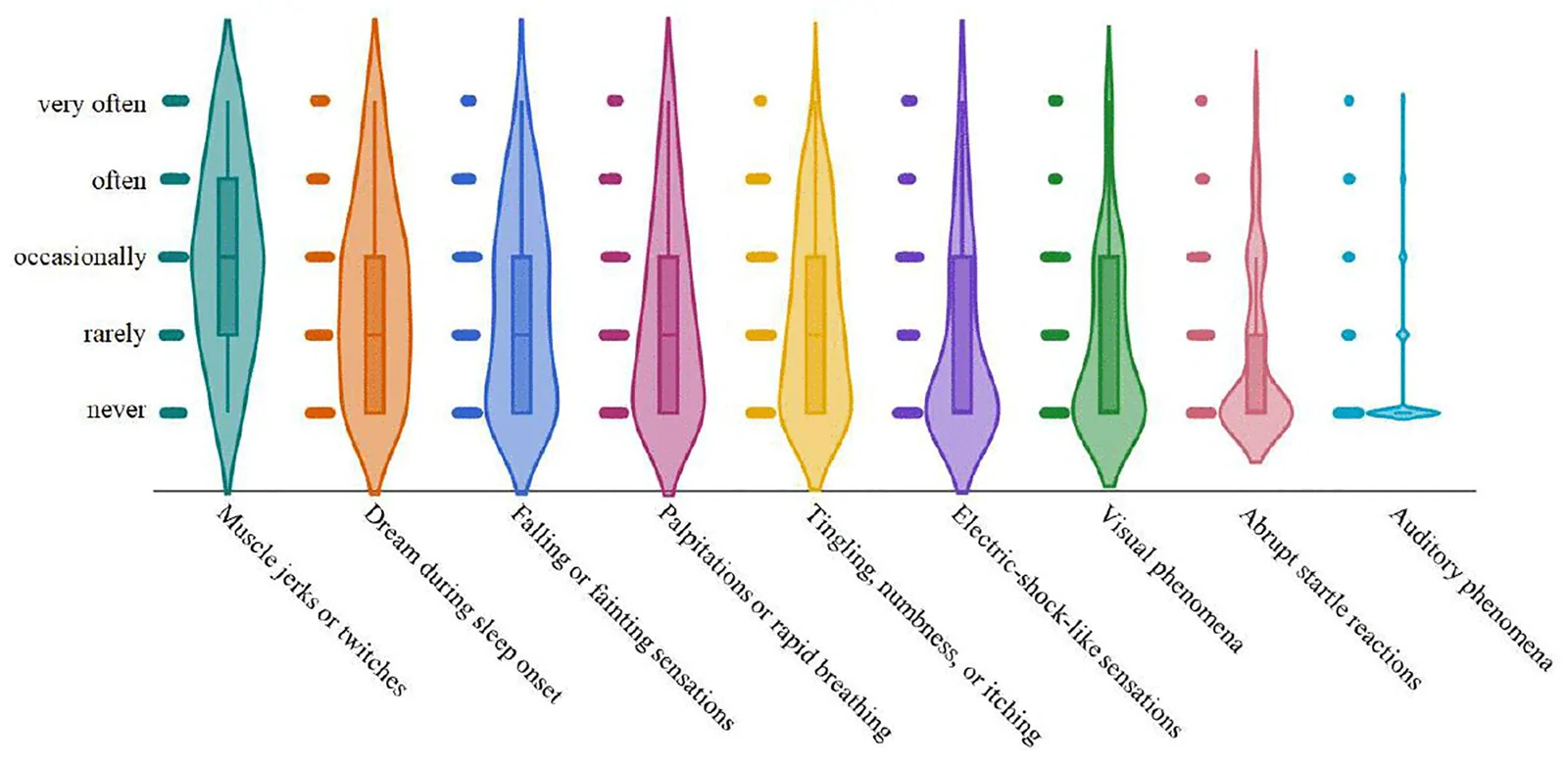
Accompanying sensations / symptoms.

**Fig 2:**
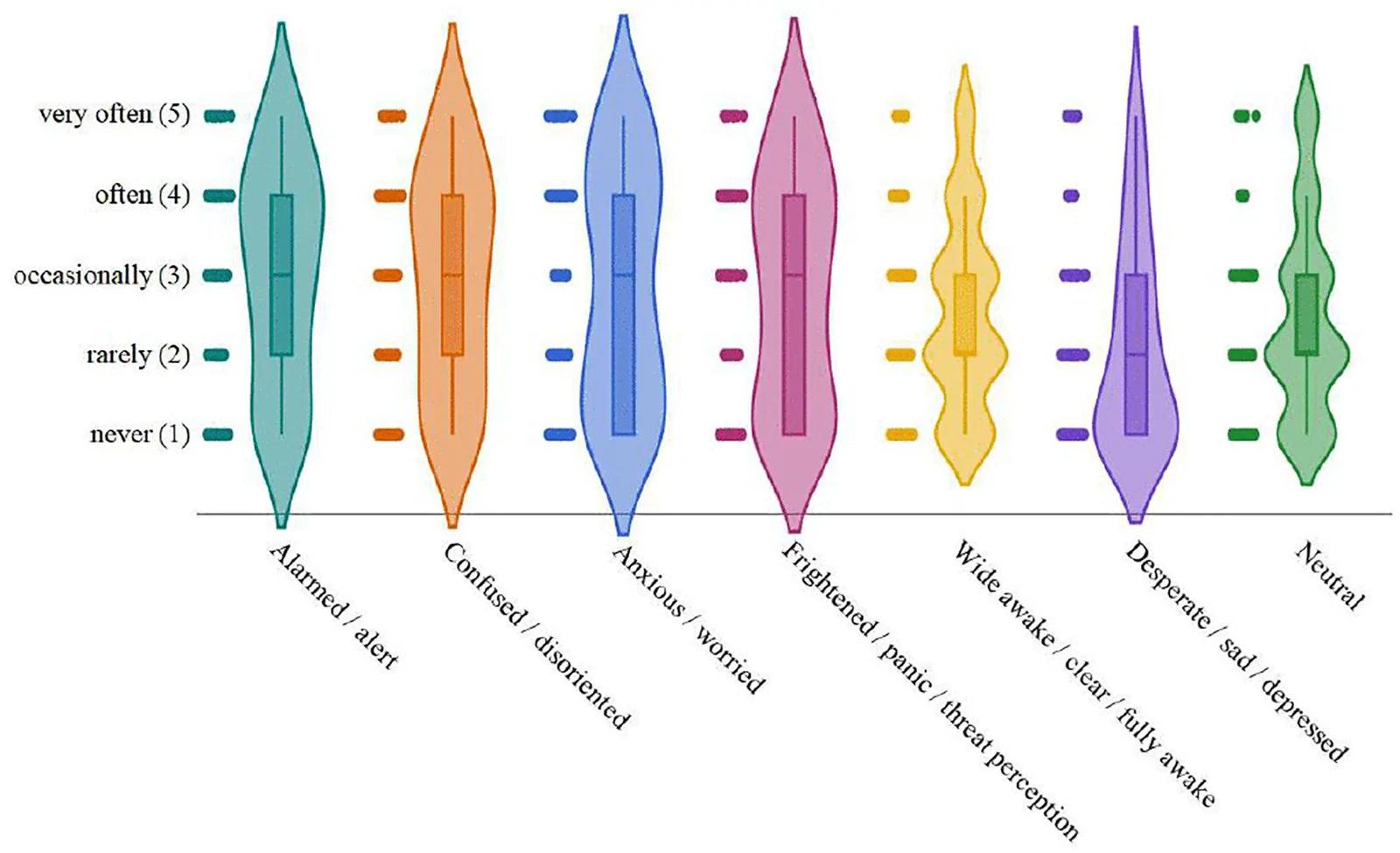
Accompanying emotions.

**Fig 3:**
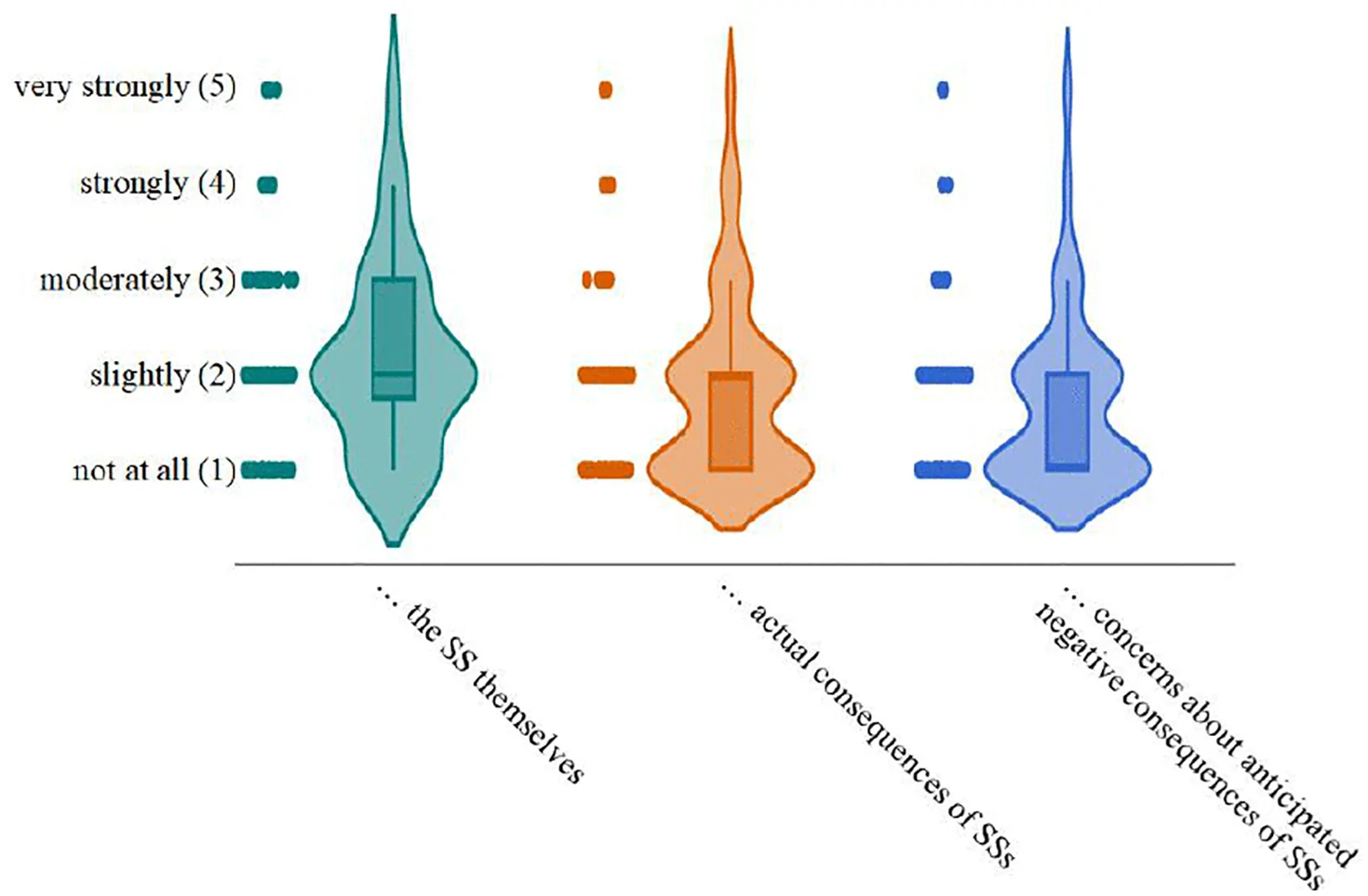
Short-term consequences.

**Fig 4:**
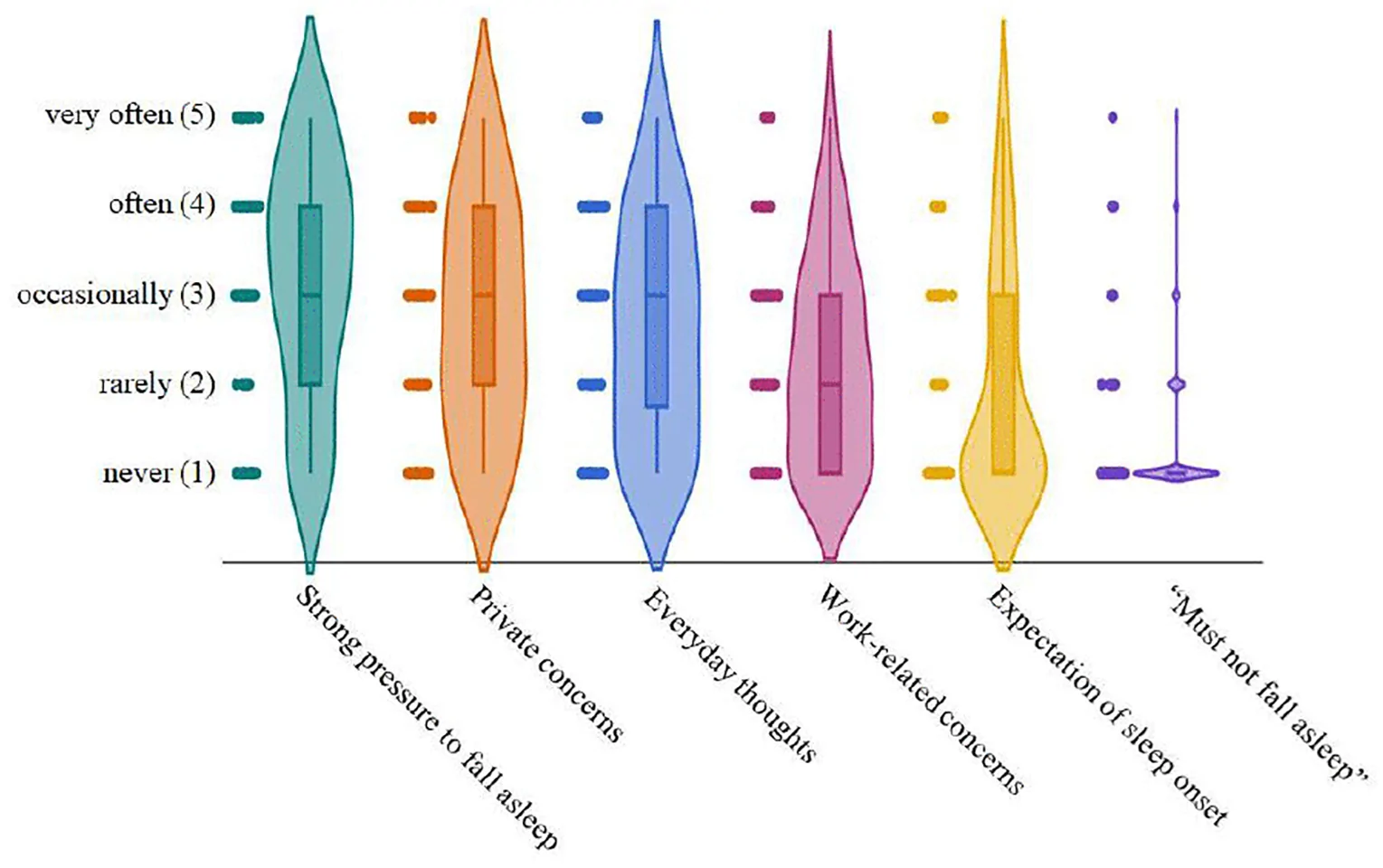
Accompanying cognitions.

**Fig 5:**
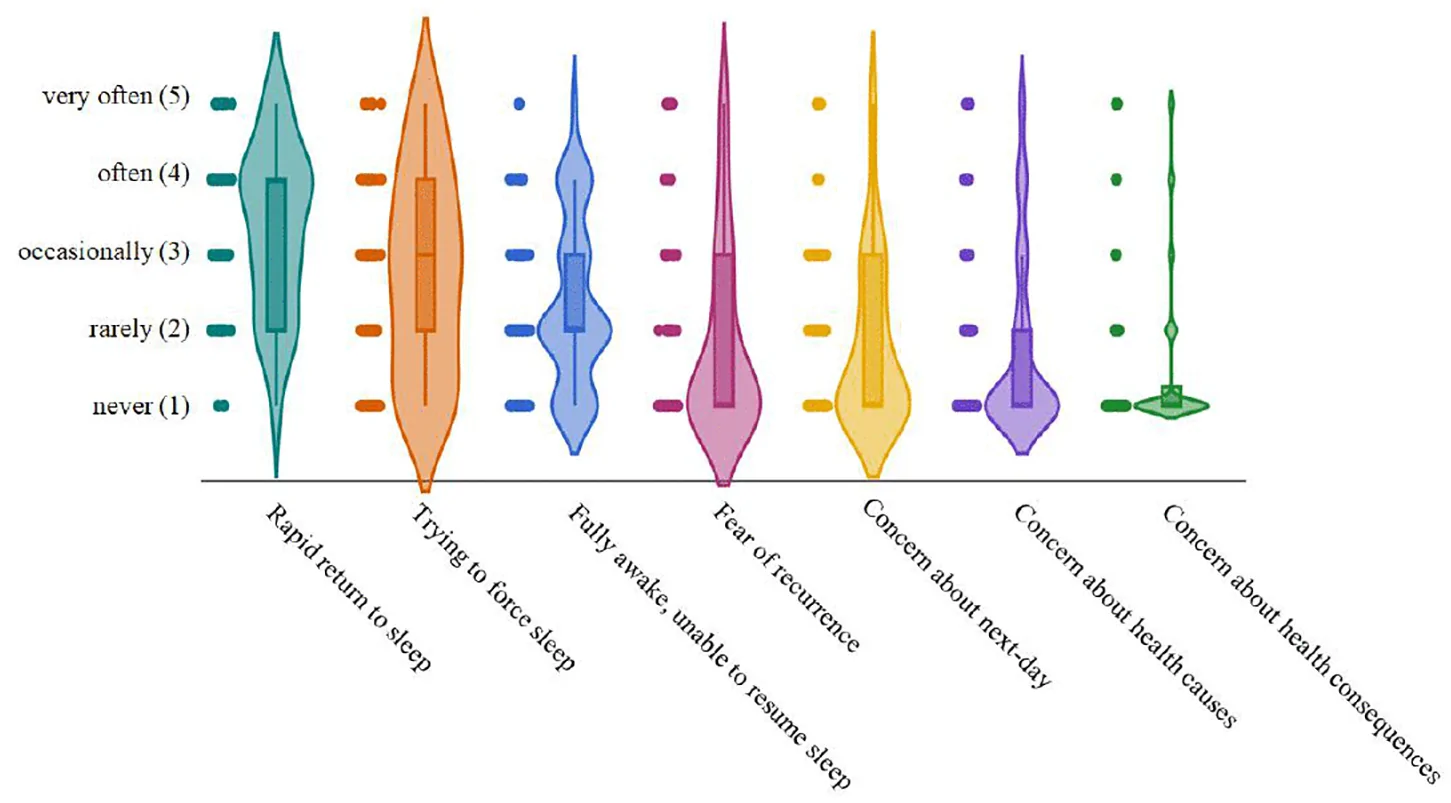
Distress related to….

**Fig 6:**
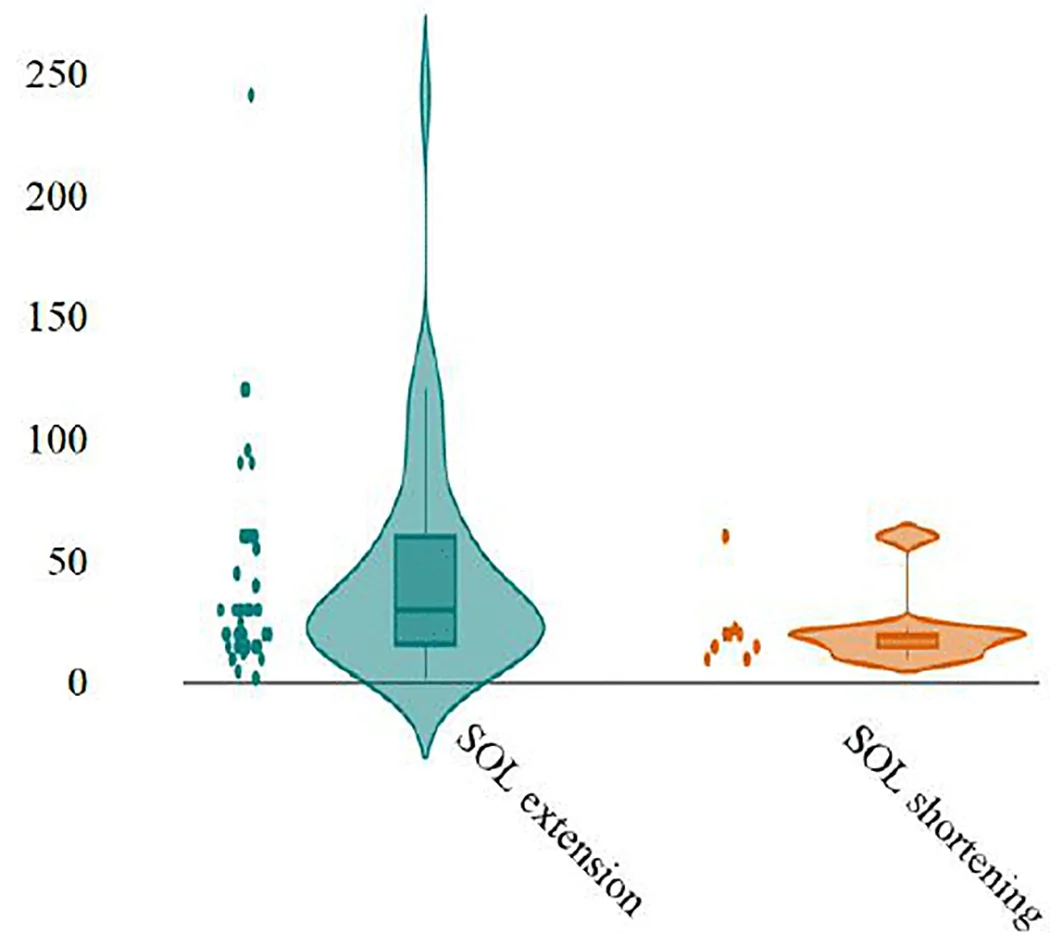
Perceived changes in sleep onset latency (SOL) due to SS.

Participants in the SS group (FoSS ≥ 1) reported a mean of 1.85 nights per week (*SD* = 1.10) with SS and 1.57 episodes per night (*SD* = 1.69). The resulting frequency index (FoSS) was *M* = 3.02 (*SD* = 3.48).

SS were characterized by a broad multimodal symptom profile including motor, sensory, emotional, cognitive, and autonomic components. Motor phenomena were most frequently reported (87.50%) and typically described as sudden muscle jerks or twitches affecting the limbs, head, or whole body, occasionally leading to abrupt awakening.

Sensory phenomena were also common and included sensations of falling or loss of stability (65.62%) as well as somatosensory experiences such as tingling, numbness, or pins-and-needles sensations (63.54%). Electric-shock-like sensations were reported by 44.79% of participants. Visual phenomena (47.92%) included flashes or flickering with closed eyes, while auditory phenomena (21.88%) involved internally perceived loud or explosive sounds. SS were frequently accompanied by autonomic arousal, including palpitations, increased heart rate, or rapid breathing (62.50%). A substantial proportion of participants reported dream-like experiences during sleep onset in the context of SS (73.96%), often involving falling or spatial disorientation themes.

Emotional responses were predominantly negative and arousal-based, including alarm (79.17%), confusion (79.17%), anxiety (73.96%), and panic (71.87%; acute fear with perceived immediate threat). Neutral emotional responses were also frequently endorsed (76.04%), suggesting substantial variability in subjective appraisal.

Cognitive content was characterized by sleep-related urgency (answer option: “I finally have to sleep!”; 83.33%) as well as concurrent preoccupations with private concerns, non-stressful everyday thoughts, or work-related concerns. Although most participants (95.83%) reported falling asleep again quickly, 76.04% indicated at least temporary full awakening associated with increased alertness and difficulty resuming sleep. Fear of recurrence was reported by 39.58%.

Distress related to SS was moderate on average (*M* = 2.18, *SD* = 1.00), but heterogeneous across individuals. Approximately half of participants reported distress related to actual (54.17%) or anticipated negative consequences of SS (47.92%). Effects on sleep-onset latency varied, with 51.04% reporting prolongation, 9.38% reporting shortening, and 40.63% reporting no change. SS were perceived as more frequent under conditions of psychological stress, tension, and emotional burden, whereas relaxation was associated with reduced frequency. Sleep deprivation was less consistently endorsed as a modulating factor.

Participants reported a range of coping strategies, most commonly deliberately redirecting thoughts at sleep onset, relaxation techniques, exercise, and reduction of sleep-environmental disturbances (e.g., noise or light). Pharmacological aids (sedatives, alcohol) and psychotherapy were less frequently reported but were perceived as relatively effective in the respective subset of participants.

### 3.2 Group differences and correlations

Relative to the comparison group (FoSS < 1), participants in the SS group (FoSS ≥ 1) were younger and showed poorer subjective sleep quality, lower sleep efficiency, longer sleep-onset latency, shorter total sleep time, greater insomnia severity, stronger dysfunctional sleep-related beliefs, greater perceived importance of sleep, and higher anxiety, depression, and overall psychological distress. No group difference was observed for daytime sleepiness.

Within the SS group (FoSS ≥ 1), FoSS was positively associated with poorer overall and subjective sleep quality, insomnia severity, dysfunctional sleep-related beliefs, perceived importance of sleep, anxiety, and overall psychological distress. Associations with age, sleep efficiency, sleep-onset latency, total sleep time, daytime sleepiness, and depression were not statistically significant (Table 4).

**Table 4:** Descriptive statistics, correlations with FoSS, and group differences between SS group (FoSS ≥ 1) and comparison group (FoSS < 1).

	*Groups: distribution measures*						*Correlation (df = 94)*		Mann–Whitney U test		
*Variable*	*SS group(FoSS ≥ 1, n = 96)*			*comparison group(FoSS <1, n = 98)*			*Variable, FoSS*		SS group (FoSS ≥ 1) vs. comparison group (FoSS < 1)		
	*M*	*SD*	*Md*	*M*	*SD*	*Md*	*r*_*s*_	*p*	*Z*	*p*	*r*
Age (years)	30.23	10.78	27.50	35.30	15.09	31.00	.03	.846	–2.17	.033*	.16
PSQI total	7.21	3.62	7.00	5.19	2.33	5.00	.32	.004**	–4.08	.002**	.29
SSQ	1.38	0.72	1.00	0.94	0.57	1.00	.36	.004**	–4.33	.002**	.31
efficiency %	82.89	12.27	85.71	87.98	10.67	88.89	–.07	.644	–3.20	.003**	.23
SOL (min)	33.90	28.44	30.00	25.60	25.71	15.00	.10	.453	–2.30	.028*	.17
TST (h)	6.74	1.16	7.00	7.07	1.14	7.00	–.06	.644	–2.21	.032*	.16
ISI	10.67	6.09	11.00	5.55	3.96	5.00	.34	.004**	–6.08	.002**	.44
ESS	7.89	3.78	8.00	7.06	3.90	7.00	.02	.878	–1.49	.138	
MZS	64.84	26.53	61.50	51.85	20.68	49.00	.32	.004**	–3.54	.002**	.25
HADS Anxiety	7.69	3.94	7.00	5.65	3.09	5.00	.29	.009**	–3.65	.002**	.26
HADS Depression	5.19	4.48	4.00	3.45	3.25	3.00	.17	.151	–2.65	.012*	.19
HADS total	12.88	7.43	11.00	9.10	5.58	8.00	.26	.023*	–3.68	.002**	.26
Importance of sleep	2.20	0.68	2.00	1.91	0.71	2.00	.32	.004**	–2.78	.009*	.20

p-values for correlations and Mann–Whitney U tests were adjusted for multiple comparisons using the Benjamini–Hochberg procedure.

* *p* < .05. ** *p* < .01. *** *p* < .001

### 3.3 Regression analysis

Hierarchical multiple regression analysis was conducted to examine variables jointly associated with log-transformed FoSS. In Model 1, age and gender accounted for 2.6% of the variance in FoSS (*R^2^* = .026, adjusted *R^2^* = .015), with the overall model not reaching statistical significance, *F*(2,191) = 2.51, *p* = .084. Adding PSQI total and MZS in Model 2 increased the explained variance to 24.3% (Δ*R ^2^* = .218, *F*(4,189) = 15.19, *p* < .001). The final model, additionally including HADS anxiety and depression, explained 26.9% of the variance (*R^2^* = .269, adjusted *R^2^* = .245; Δ*R^2^* = .025, *F*(6,187) = 11.45, *p* < .001). In this model, age (β = −.15, *p* = .030), PSQI total (β = .28, *p* < .001), MZS (β = .22, *p* = .004), and HADS anxiety (β = .20, *p* = .014) were significant predictors. Gender (β = .01, *p* = .896) and HADS depression (β = −.07, *p* = .437) were not significantly associated with FoSS. Thus, higher PSQI scores, stronger dysfunctional sleep-related beliefs, and higher anxiety were associated with higher FoSS, whereas higher age was associated with lower FoSS (see Table 5).

**Table 5:** Hierarchical multiple regression predicting log-transformed FoSS frequency.

*Predictor*	*B*	*SE B*	*β*	*t*	*p*	*95% CI*
**Model 1**						
**Age**	–0.01	0.004	–0.112	–1.498	.136	[–0.014, 0.002]
**Gender**	–0.14	0.125	–0.085	–1.137	.257	[–0.388, 0.104]
Model 1 statistics: *R^2^* = .026, *Adj. R^2^* = .015, *F*(2,191) = 2.51, *p* = .084						
**Model 2**						
Age	–0.01	0.004	–0.159	–2.376	.019*	[–0.016, –0.001]
Gender	–0.03	0.111	–0.020	–0.306	.760	[–0.254, 0.186]
PSQI total	0.07	0.016	0.298	4.216	< .001***	[0.035, 0.097]
MZS total	0.01	0.002	0.260	3.689	< .001***	[0.004, 0.012]
Model 2 statistics: *R^2^* = .243, *Adj. R^2^* = .227, Δ*R^2^* = .218, *F*(4,189) = 15.192, *p* < .001						
**Model 3**						
Age	–0.008	0.004	–0.146	–2.186	.030*	[–0.015, –0.001]
Gender	0.015	0.113	0.009	0.131	.896	[–0.208, 0.238]
PSQI total	0.062	0.016	0.277	3.797	< .001***	[0.030, 0.094]
MZS total	0.006	0.002	0.218	2.931	.004**	[0.002, 0.011]
HADS Anxiety	0.039	0.016	0.202	2.489	.014*	[0.008, 0.070]
HADS Depression	–0.012	0.015	–0.065	–0.779	.437	[–0.041, 0.018]
Model 3 statistics: *R^2^* = .269, *Adj. R^2^* = .245, Δ*R²* = .025, *F*(6,187) = 11.446, *p* < .001						

**Note.** FoSS was log-transformed (ln(FoSS + 1)) prior to analysis due to positive skewness.* *p* < .05. ** *p* < .01. *** *p* < .001

### 3.4 Exploratory analyses

Women were significantly overrepresented in the SSs group (FoSS ≥ 1) compared with the FoSS < 1 group, χ^2^(1) = 5.21, *p* = .022, *Cramer’s V* = .164. Participants in the SS group (FoSS ≥ 1) reported higher rates of sleep-related conditions, particularly sleep disorders (χ^2^(1) = 10.08, *p* = .001, *Cramer’s V* = .228) and nightmares (χ^2^(1) = 8.01, *p* = .005, *Cramer’s V* = .203).No group differences were observed for anxiety, depression, or PTSD diagnoses.

Those in the SS group (FoSS ≥ 1) further reported higher levels of personal concerns χ^2^(1) = 7.69, *p* = .006, *Cramer’s V* = .199, whereas no group differences emerged for occupational concerns or substance use. Full contingency tables are provided in the S1 Appendix.

### 3.5 Sensitivity analyses

Sensitivity analyses using the 25^th^ (FoSS = / ≠ 0.00) and 75^th^ (FoSS < / ≥ 2.00) percentiles as alternative cut-offs yielded largely consistent patterns of group differences. Significant group differences for PSQI total, SSQ, sleep efficiency, sleep-onset latency (SOL), ISI, MZS, HADS anxiety, HADS total, and importance of sleep were observed across all three threshold definitions, with effect sizes remaining comparable across cut-offs (*r* = .16 to  .45). In contrast, the group difference in total sleep time (TST) observed with the post hoc cut-off was no longer significant with either percentile-based cut-off. The group difference in age was significant with the post hoc and 25^th^-percentile cut-offs but not with the 75^th^-percentile cut-off. For HADS depression, the group difference was significant with the post hoc and 75^th^-percentile cut-offs but not with the 25^th^-percentile cut-off.

For the correlations, the findings were more variable across threshold definitions. Significant positive correlations with PSQI total, MZS, and importance of sleep were observed across all three threshold definitions, with comparable correlation coefficients (PSQI total: *r_s_* = .28 to  .34; MZS: *R_s_* = .32 to  .37; importance of sleep: *R_s_* = .29 to.41). The positive correlation with SSQ was observed across all three thresholds (*R_s_* = .20 to  .36), although it was no longer significant with ^th^e 75^th^-percentile cut-off. In contrast, correlations with ISI, HADS anxiety, and HADS total were significant with ^th^e post hoc and 25^th^-percentile cut-offs but not with ^th^e 75^th^-percentile cut-off. The correlation with HADS depression was significant only with ^th^e 25^th^-percentile cut-off. No significant correlations with age, sleep efficiency, SOL, TST, or ESS were observed across the three threshold definitions (Table 6).

**Table 6:** Sensitivity analyses of group differences.

	*Group differences*			*Correlations*		
*Variable*	*Post hoc cut-offFoSS < / ≥ 1.00*	*25*^*th*^ *percentileFoSS = / ≠ 0.00*	*75*^*th*^ *percentileFoSS < / ≥ 2.00*	*Post hoc cut-offFoSS < / ≥ 1.00*	*25*^*th*^ *percentileFoSS = / ≠ 0.00*	*75*^*th*^ *percentileFoSS < / ≥ 2.00*
**Age**	*p* =.033, r =.16	p=.047, r =.15	p= .091, r =.13	*r*_*s*_ = .03, *p* = .846	*r*_*s*_= −.05, *p* =.657	*r*_*s*_= .17, *p* =.220
PSQI total	*p* = .002, *r* = .29	*p* = .004, *r* =.25	*p* = .002, *r* =.33	*r*_*s*_ =.32, *p* = .004	*r*_*s*_= .34, *p* = .002	*r*_*s*_=.28, *p* = .039
SSQ	*p* = .002, *r* =.31	*p* = .004, *r* =.32	*p* = .002, *r* =.37	*r*_*s*_ =.36, *p* = .004	*r*_*s*_= .33, *p* = .002	*r*_*s*_=.20, *p* = .383
Sleep efficiency	*p* = .003, *r* =.23	*p* = .009, *r* =.21	*p* = .032, *r* =.16	*r*_*s*_ = −.07, *p* = .644	*r*_*s*_= −.13, *p* =.250	*r*_*s*_= −.20, *p* =.145
SOL	*p* = .028, *r* =.17	*p* = .017, *r* =.18	*p* = .010, *r* =.20	*r*_*s*_ = .10, *p* = .453	*r*_*s*_= .13, *p* =.250	*r*_*s*_= −.02, *p* =.913
TST	*p* = .032, *r* =.16	*p* = .129, *r* =.12	*p* = .157, *r* =.11	*r*_*s*_ = −.06, *p* = .644	*r*_*s*_= −.10, *p* =.358	*r*_*s*_= −.09, *p* =.497
ISI	*p* = .002, *r* =.44	*p* = .004, *r* =.43	*p* = .002, *r* =.45	*r*_*s*_ = .34, *p* = .004	*r*_*s*_= .37, *p* = .002	*r*_*s*_= .09, *p* =.499
ESS	*p* = .138	*p* = .172	*p* = .597	*r*_*s*_ = .02, *p* = .878	*r*_*s*_= .01, *p* =.935	*r*_*s*_= .16, *p* =.237
MZS	*p* = .002, *r* =.25	*p* = .007, *r* =.22	*p* = .002, *r* =.34	*r*_*s*_ = .32, *p* = .004	*r*_*s*_= .37, *p* = .002	*r*_*s*_= .33, *p* = .015
HADS Anxiety	*p* = .002, *r* =.26	*p* = .009, *r* =.21	*p* = .002, *r* =.33	*r*_*s*_ = .29, *p* = .009	*r*_*s*_= .34, *p* = .002	*r*_*s*_= .03, *p* =.859
HADS Depression	*p* = .012, *r* =.19	*p* = .140	*p* = .015, *r* =.18	*r*_*s*_ = .17, *p* = .151	*r*_*s*_= .24, *p* =.022	*r*_*s*_= .08, *p* =.545
HADS total	*p* = .002, *r* =.26	*p* = .015, *r* =.19	*p* = .002, *r* =.29	*r*_*s*_ = .26, *p* =.023	*r*_*s*_= .32, *p* =.002	*r*_*s*_= .04, *p* =.786
Importance of sleep	*p* = .009, *r* =.20	*p* = .015, *r* =.19	*p* = .002, *r* =.25	*r*_*s*_ = .32, *p* = .004	*r*_*s*_= .29, *p* = .004	*r*_*s*_= .41, *p* = .002

p-values for correlations and Mann–Whitney U tests were adjusted for multiple comparisons using the Benjamini–Hochberg procedure.

* *p* < .05. ** *p* < .01. *** *p* < .001

Full results of the sensitivity analyses are provided in S1 Appendix.

## 4 Discussion

This study used a mixed-methods approach to examine the phenomenology of sleep starts (SS) and their associations with sleep-related and psychological variables. Overall, SS emerged as a heterogeneous phenomenon extending beyond isolated motor jerks and frequently involving emotional, cognitive, autonomic, and perceptual experiences. While many participants described SS as familiar or only mildly impairing, a substantial subgroup reported marked distress, repeated sleep disruption, prolonged sleep-onset latency, and elevated psychological burden.

SS were experienced as multimodal events including motor, sensory, emotional, cognitive, and autonomic components. Motor phenomena were most frequently reported, typically described as sudden jerks or twitches affecting one or more limbs or the whole body and occasionally leading to abrupt awakening. These motor events often co-occurred with falling- or imbalance-related dream content, suggesting overlap with unstable sleep-onset transitions [1]. However, the present cross-sectional design does not permit conclusions about temporal precedence or causal relationships.

Sensory and perceptual experiences were also common, including sensations of falling, somatosensory phenomena, visual flashes, and auditory distortions. A substantial proportion of participants also reported dream-like experiences during SS at sleep onset, often involving falling or spatial disorientation themes. These findings suggest overlap with other sleep-onset phenomena such as hypnagogic hallucinations and related states. However, these could not be reliably differentiated in the present design despite attempts to capture symptom-specific characteristics. Emotional responses were predominantly arousal-based and negative, including alarm, confusion, anxiety, and panic (defined here as acute fear with perceived immediate threat). Neutral emotional experiences were also frequently endorsed, indicating substantial variability in subjective appraisal.

A central finding was the prominent role of physiological and emotional arousal. Participants frequently reported autonomic activation (e.g., palpitations, increased heart rate, rapid breathing) as well as heightened emotional arousal (alarm, confusion, anxiety, panic). This pattern aligns with the common characterization of insomnia as a “hyperarousal disorder” [28,29], reflecting heightened physiological and cognitive-emotional activation that can interfere with the initiation of sleep. This interpretation is supported by group differences and correlational findings.

Compared with controls, participants with SS (FoSS ≥ 1) showed poorer overall (PSQI total) and subjective (SSQ) sleep quality, lower sleep efficiency, longer sleep-onset latency, shorter total sleep time, greater insomnia severity, stronger dysfunctional sleep-related beliefs, greater perceived importance of sleep, and elevated anxiety, depression, and overall psychological distress. Within the SS group (FoSS ≥ 1), SS frequency was positively associated with poorer sleep quality, higher insomnia severity, stronger dysfunctional sleep-related beliefs, greater perceived importance of sleep, and higher anxiety and overall psychological distress. In contrast, associations with sleep efficiency, sleep-onset latency, total sleep time, daytime sleepiness, and depression were not statistically significant. Together, these findings suggest that SS frequency is more closely linked to subjective sleep appraisal and cognitive-emotional activation than to sleep quantity or daytime sleepiness per se.

Participants who placed greater importance on sleep reported more frequent SS, and SS were also associated with stronger dysfunctional sleep-related beliefs. Sleep valuation and dysfunctional sleep-related beliefs were themselves moderately correlated (*r* = .44, *p* < .001).

The sensitivity analyses provide support for the robustness of several main group differences across alternative threshold definitions. In particular, group differences in PSQI total, SSQ, sleep efficiency, SOL, ISI, MZS, HADS anxiety, HADS total, and importance of sleep remained significant across all three cut-offs with comparable effect sizes. In contrast, the group difference in TST was not maintained under either alternative cut-off, while the findings for age and HADS depression varied depending on the threshold. The correlational findings were more heterogeneous. Associations of FoSS with PSQI total, MZS, and importance of sleep were consistently observed across threshold definitions, whereas associations with ISI, HADS anxiety, HADS depression, and HADS total were sensitive to the applied cut-off. The association with SSQ showed a consistent direction but was no longer statistically significant at the 75^th^-percentile cut-off. These findings suggest that the main group-level differences are comparatively robust to variation in group classification, whereas some associations between SS frequency and individual characteristics may depend on the operational definition of the SS group.

Regression analyses further indicated that poorer overall sleep quality (PSQI total) and stronger dysfunctional sleep-related beliefs (MZS) were independently associated with higher FoSS after adjustment for age and gender. Adding PSQI and MZS to the demographic model increased the explained variance from 2.6% to 24.3%. The addition of anxiety and depression resulted in a further increase to 26.9%, with anxiety remaining a significant predictor in the final model, whereas depressive symptoms did not. Age was also a significant predictor in the final model, with higher age associated with lower FoSS, whereas gender was not. These findings indicate that the associations of sleep quality, dysfunctional sleep-related beliefs, and anxiety with FoSS were not fully accounted for by the other variables included in the model.

This pattern of findings, particularly the independent associations of sleep quality, dysfunctional sleep-related beliefs, and anxiety with FoSS, is consistent with cognitive models of insomnia, particularly the attention–intention–effort pathway [30], which proposes that increased sleep-related monitoring and effortful attempts to initiate sleep can paradoxically increase cognitive and emotional arousal, thereby interfering with sleep onset. Participants frequently reported self-imposed pressure to fall asleep and cognitive preoccupation during SS, including private concerns, non-stressful everyday thoughts, and work-related concerns. Moreover, dysfunctional sleep-related beliefs and the perceived importance of sleep were associated with FoSS. Taken together, these findings suggest that SS occur within a broader context of sleep-related cognitive-emotional activation, where increased monitoring and concern about sleep may heighten awareness of internal sleep-onset phenomena and reinforce anticipatory worry. This aligns with prior assumptions regarding stress-related modulation of SS [1,2] and provides additional support for the role of heightened emotional and cognitive arousal. However, these data are based on retrospective self-report and cannot establish causality.

SS were perceived as more frequent during psychological stress, tension, and emotional burden, whereas relaxation was associated with reduced frequency. In contrast, sleep deprivation and microsleep-related tendencies were less consistently endorsed as modulators.

As self-initiated strategies for reducing the frequency of SS, participants most commonly reported deliberately redirecting their thoughts during sleep onset, alongside relaxation techniques, physical exercise, and reducing environmental disturbances. These strategies were generally perceived as having limited effects on SS frequency. Pharmacological aids and psychotherapy were less frequently used but were perceived as relatively more effective in a subset of participants. The prominence of arousal-reducing strategies aligns with clinical approaches used in insomnia treatment, particularly cognitive behavioral therapy for insomnia (CBT-I; [31], and with hyperarousal-based models of insomnia [2,29,32], as outlined above.

Although SS were associated with increased distress in a substantial subgroup, mean distress levels due to SS were moderate and highly variable across individuals. Many participants reported rapid return to sleep and limited impairment, whereas others experienced repeated awakenings, prolonged sleep-onset latency, and marked distress. This heterogeneity suggests that SS range from benign transitional phenomena to clinically relevant sleep-disruptive experiences depending on frequency, appraisal, and associated emotional response. Demographically, SS were more common in younger individuals and in women. The age effect contrasts with typical patterns in sleep disorders [33,34] and may indicate greater vulnerability to SS phenomena in younger individuals, although sampling or self-selection effects cannot be ruled out. Notably, younger age also remained independently associated with higher FoSS in the final regression model after adjustment for sleep-related and psychological variables. The gender difference was small but consistent, suggesting a modest overrepresentation of women in the SS group (FoSS ≥ 1) without clear evidence for a strong gender-specific effect.

Participants with SS (FoSS ≥ 1) differed from controls across multiple domains, including sleep quality, sleep onset latency, sleep efficiency, insomnia severity, dysfunctional sleep-related beliefs, perceived importance of sleep, anxiety, depression, and overall psychological distress. Within the SS group, FoSS showed associations with several of these variables, particularly subjective sleep quality, dysfunctional sleep-related beliefs, and perceived importance of sleep. However, the sensitivity analyses indicated that some associations between FoSS and psychological variables were dependent on the applied threshold. Overall, SS appear more strongly related to subjective sleep appraisal and cognitive-emotional factors than to sleep duration or daytime sleepiness.

Chronotype was not explicitly assessed in the present study to reduce participant burden in an already extensive questionnaire. While habitual sleep timing variables from the PSQI may reflect aspects of sleep behavior, they are strongly influenced by social and occupational constraints and do not adequately capture endogenous circadian preference. Accordingly, these variables were not considered a valid proxy for chronotype. Although circadian preference may plausibly influence sleep-onset processes, there is currently no empirical evidence linking chronotype to SS. Future studies should explicitly address this potential factor.

Exploratory analyses further indicated higher rates of sleep disorders and nightmares in the SS group (FoSS ≥ 1), but no consistent differences for other clinical conditions.

### 4.1 Limitations

Several limitations should be considered. The cross-sectional design and reliance on retrospective self-report preclude causal inference. No objective sleep measures were included. The sample was not stratified, and recruitment targeted individuals with and without SS, limiting generalizability and preventing prevalence estimation. Phenomenological items were self-developed and could not be psychometrically validated against an established measure because no validated instrument assessing SS phenomenology currently exists.

All analyses were exploratory and hypothesis-generating. The regression analyses should likewise be interpreted as exploratory associations rather than evidence of temporal or causal relationships, particularly given the cross-sectional design and the number of variables considered.

A limitation of the present study is that the threshold used to define the SS group was determined post hoc and in a data-driven manner. Sensitivity analyses using the 25^th^ and 75^th^ percentiles as alternative cut-offs showed that several of the main group differences, particularly those involving sleep quality, sleep efficiency, sleep-onset latency, insomnia severity, dysfunctional sleep-related beliefs, anxiety, overall psychological distress, and importance of sleep, were robust across threshold definitions. However, the group difference in total sleep time was not replicated under either alternative cut-off, and the findings for age and depression varied across thresholds. Correlational findings were more threshold-sensitive, particularly for insomnia severity and anxiety-related measures. Thus, these associations should be interpreted with caution and require confirmation in future studies using prospectively defined and independently validated criteria for SS classification.

### 4.2 Strengths

Despite these limitations, this study provides one of the first larger mixed-methods investigations of SS combining quantitative and qualitative data. The inclusion of a control group allowed systematic comparison across sleep and psychological domains. The findings extend prior work by demonstrating that SS involve multimodal subjective experiences beyond motor phenomena.

## 5 Conclusion and outlook

SS represent heterogeneous sleep-onset phenomena involving motor, emotional, cognitive, and perceptual experiences. While often benign, they may be associated with clinically relevant distress and sleep disruption in a subgroup of individuals. SS frequency is most consistently associated with subjective sleep quality, dysfunctional sleep-related beliefs, and anxiety symptoms. These findings support the view that SS should be considered within broader frameworks of sleep-related hyperarousal and cognitive-emotional activation. Future longitudinal and experimental studies incorporating objective sleep measures are needed to clarify temporal dynamics and mechanisms. Interventions targeting arousal regulation, maladaptive sleep-related cognitions, and stress may represent promising directions for future research and clinical practice.

## Supporting information

S1 Data This SPSS dataset contains the minimal data required to replicate all results reported in the manuscript, including the values underlying statistical analyses, tables, and figures. (SAV)

S1 Appendix Table A. Sample descriptions and distribution measures. **Table B.** Sleep start phenomenology and associated experiences. **Fig A.** Conditions subjectively associated with changes in FoSS. **Fig B.** Self-initiated countermeasures and perceived changes in FoSS. **Table C.** Frequencies: Which sensations/symptoms were experienced how often by how many participants (multiple answers)? **Table D.** Frequencies of accompanying emotions/feelings (multiple answers). **Table E.** Frequencies of short-term consequences (multiple answers). **Table F.** Frequencies of accompanying cognitions (multiple answers). **G.** Open-text responses to the “Other” option for individual symptom descriptions and associated experiences. **Table H.** Conditions subjectively influencing the FoSS (multiple answers). **Table I.** Countermeasures (multiple answers). **Table J.** Efficacy of countermeasures (multiple answers). **Table K.** Pre-existing physical illnesses, mental disorders, and substance use. **Table L.** Personal and occupational concerns. **Table M.** Sensitivity analysis, 25^th^ percentile. **Table N.** Sensitivity analysis, 75^th^ percentile. **O.** Instrument: Online-survey.
(PDF)
