# Supplementary material for: Sleep starts: Phenomenology and associations with sleep, psychological, and demographic characteristics
Source: PLOS Ment Health. 2026 Nov 11;3(11):e0000737. doi: 10.1371/journal.pmen.0000737 (PMC13754169; doi:10.1371/journal.pmen.0000737)
Supplement: Supplementary file 1 [file pmen.0000737.s002.pdf]

S1 Appendix

Supporting Information

Sleep Starts: Phenomenology and Associations with Sleep, Psychological, and Demographic Characteristics.

A Mixed-Methods Online Survey of Sleep Starts and Their Associations with Sleep and Psychological Characteristics

Tanja Grünberger, Christopher Höhn, Manuel Schabus, Anton-Rupert Laireiter

Department of Psychology, Paris-Lodron-University Salzburg, Salzburg, Austria

Table A. Sample descriptions and distribution measures.

Variables	N_{total} = 194				$n_{SS\ group\ (FoSS \geq 1)}$ = 96				$n_{comparison\ group\ (FoSS < 1)}$ = 98			
	M	*SD*	*Md*	*range*	*M*	*SD*	*Md*	*range*	*M*	*SD*	*Md*	*range*
PSQI total	6.19	3.19	6.00	1–17	7.21	3.62	7.00	1–17	5.19	2.33	5.00	1–15
SSQ	1.15	0.68	1.00	0–3	1.38	0.72	1.00	0–3	0.94	0.57	1.00	0–2
Efficiency (%)	85.46	11.74	87.50	42.86–100.00	82.89	12.27	85.71	44.44–100.00	87.98	10.67	88.89	42.86–100.00
SOL (min)	29.71	27.35	20.00	1–120	33.90	28.44	30.00	1–120	25.60	25.71	15.00	1–120
TST (h)	6.91	1.16	7.00	3.00–9.00	6.74	1.16	7.00	4.00–9.00	7.07	1.14	7.00	3.00–9.00
ISI	8.08	5.72	7.00	0–26	10.67	6.09	11.00	0–26	5.55	3.96	5.00	0–16
ESS	7.47	3.85	7.00	0–19	7.89	3.78	8.00	0–19	7.06	3.90	7.00	0–18
MZS	58.28	24.58	56.00	6–135	64.84	26.53	61.50	6–135	51.85	20.68	49.00	7–111
HADS Anxiety	6.66	3.67	6.00	0–20	7.69	3.94	7.00	1–20	5.65	3.09	5.00	0–13
HADS Depression	4.31	3.99	3.00	0–18	5.19	4.48	4.00	0–18	3.45	3.25	3.00	0–17
HADS total	10.97	6.81	9.00	0–36	12.88	7.43	11.00	1–36	9.10	5.58	8.00	0–29

Table B. Self-reported conditions and countermeasures associated with perceived changes in FoSS

**Conditions subjectively associated with changes in FoSS**				
1 = much less frequent, 2 = less frequent, 3 = no change, 4 = more frequent, 5 = much more frequent.	*M (95% CI)*	*SD*	*Md*	*n (%) ≠ 3*
When feeling mentally unwell	3.70 (3.54–3.86)	0.80	4.00	62 (64.58%)
When feeling tense / stressed	3.64 (3.47–3.80)	0.82	4.00	61 (63.54%)
During private concerns	3.61 (3.47–3.76)	0.72	4.00	58 (60.42%)
During work-related concerns	3.38 (3.24–3.51)	0.68	3.00	43 (44.79%)
Under unusual sleeping conditions	3.18 (2.97–3.39)	1.04	3.00	51 (53.12%)
When feeling physically unwell	3.13 (2.97–3.28)	0.77	3.00	37 (38.54%)
After sleep deprivation / poor night	3.07 (2.88–3.26)	0.94	3.00	43 (44.79%)
Microsleep tendency / dozing off	2.79 (2.63–2.96)	0.82	3.00	32 (33.33%)
When feeling relaxed / balanced	2.19 (2.02–2.36)	0.85	2.00	60 (62.50%)
**Self-initiated countermeasures and perceived changes in FoSS**				
0 = not tried, 1 = much less frequent, 2 = less frequent, 3 = no change, 4 = more frequent, 5 = much more frequent.	*M (95% CI)*	*SD*	*Md*	*n (%) ≠ 0*
Psychotherapy	2.67 (2.33–3.00)	0.62	3.00	15 (15.62%)
Trying to redirect thoughts	2.63 (2.41–2.85)	0.91	3.00	68 (70.83%)
Exercise / sports	2.61 (2.37–2.85)	0.94	3.00	61 (63.54%)
Relaxation exercises	2.60 (2.36–2.83)	0.88	3.00	57 (59.37%)
Reduce external disturbances	2.59 (2.34–2.84)	0.86	3.00	49 (51.04%)
Alcohol to help fall asleep	2.42 (2.06–2.79)	0.90	3.00	26 (27.08%)
Sleep aids / sedatives	2.09 (1.62–2.56)	1.06	2.00	22 (22.08%)

Note. Means, standard deviations, medians, and confidence intervals were calculated only for participants who reported having tried the respective countermeasure (ratings 1–5). The value 0 ("not tried") was used exclusively to determine the proportion of participants who had attempted the respective strategy.
n (%) ≠ 3: number and percentage of participants in the SS group reporting a change from the baseline frequency (response score ≠ 3); scores 1–2 indicate a decrease in frequency, whereas scores 4–5 indicate an increase in frequency.
n (%) ≠ 0: number and percentage of participants in the SS group reporting having tried the respective symptom or behavior at least once (response score ≠ 0).

Figure A. Conditions subjectively associated with changes in FoSS

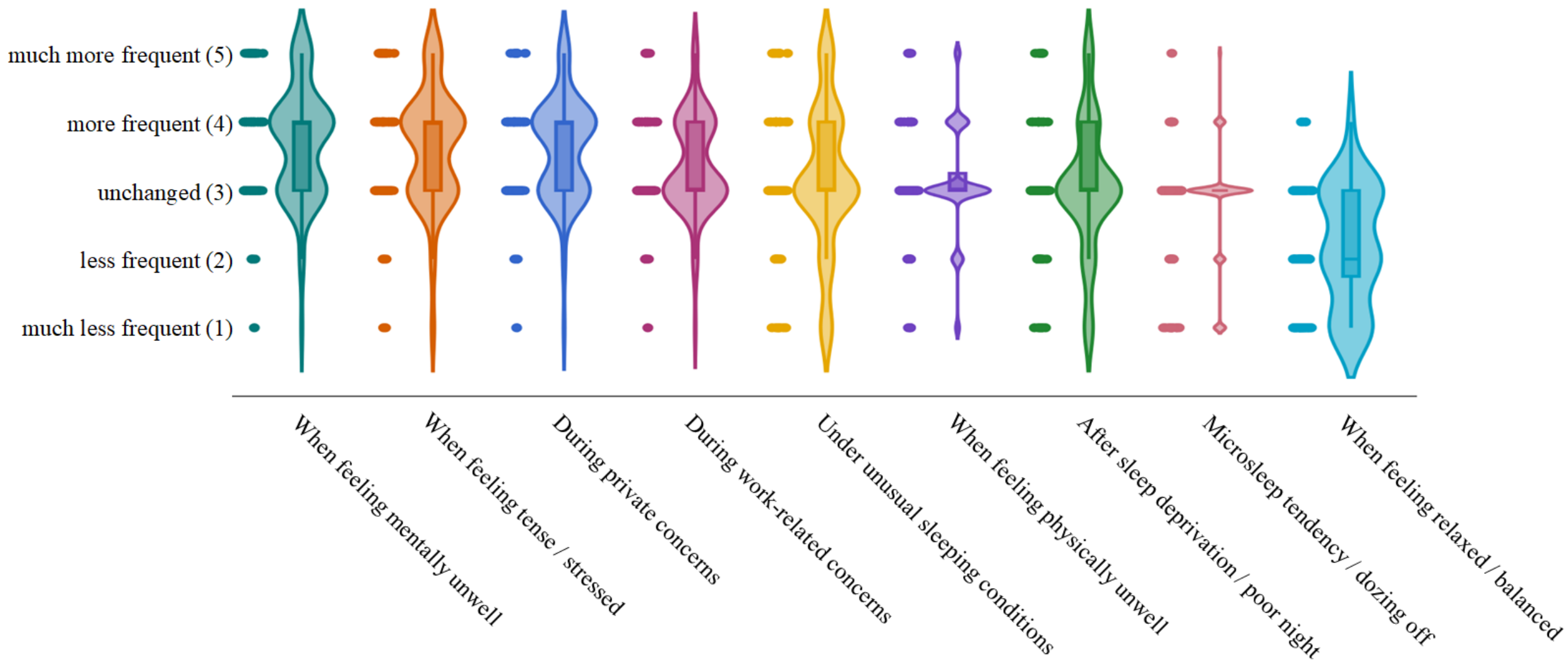

Figure B. Self-initiated countermeasures and perceived changes in FoSS

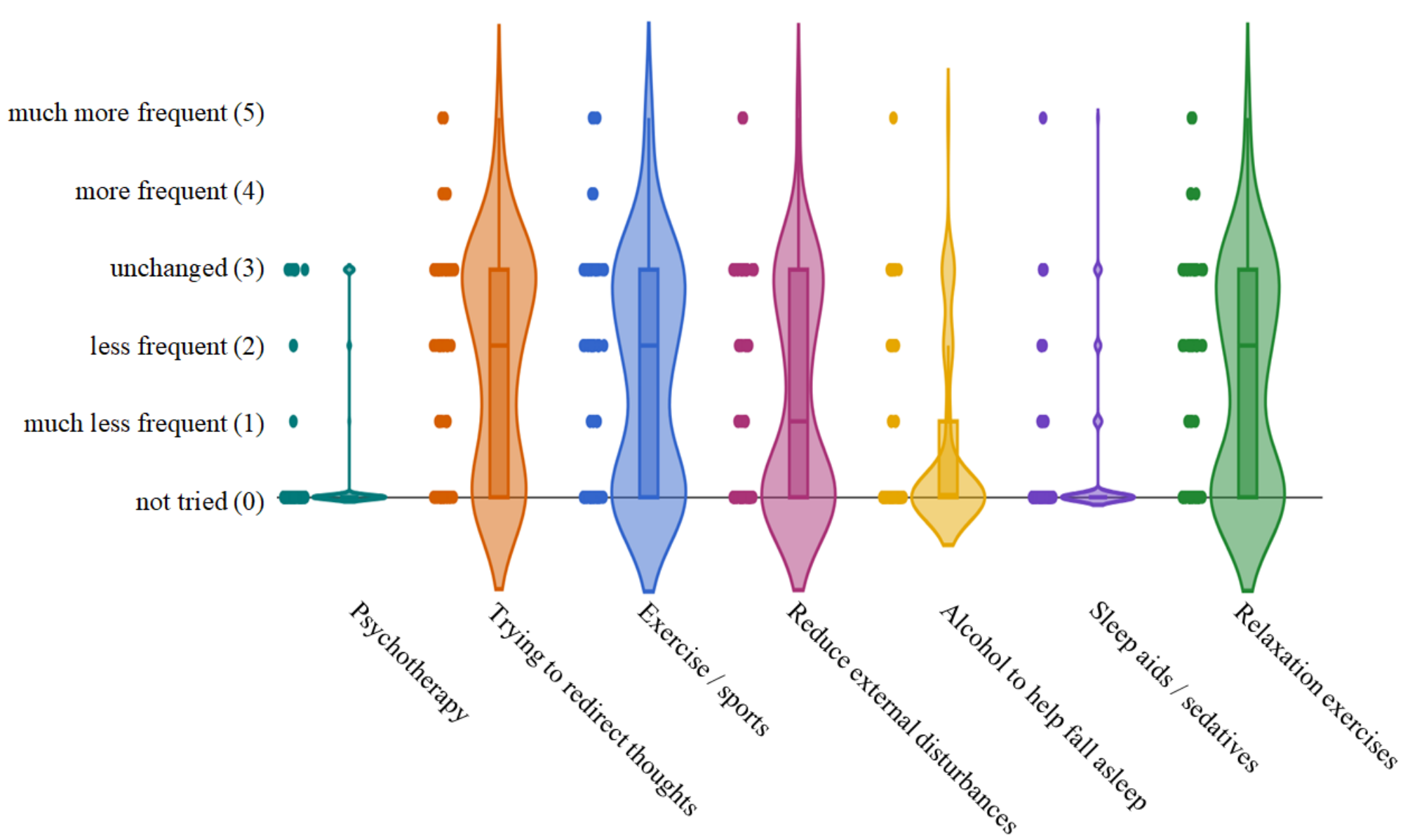

Table C. Frequencies: Which sensations/symptoms were experienced how often by how many participants (multiple answers)?

How often do you experience something from the following list when SS occur while falling asleep?	*M*	*SD*	*Md*		*never (1)*	*sporadically (2)*	*occasionally (3)*	*often (4)*	*very often (5)*
muscle twitches, e.g. in legs, arms or head	2.97	1.21	3.00	*n*	12	23	29	20	12
				%	12.50	23.96	30.21	20.83	12.50
dreams, even at the falling asleep	2.49	1.22	2.00	*n*	25	26	25	13	7
				%	26.04	27.08	26.04	13.54	7.29
other sensations, e.g. feeling of falling or fainting	2.33	1.22	2.00	*n*	33	21	23	15	4
				%	34.38	21.88	23.96	15.63	4.17
heart palpitations, increased pulse, rapid breathing	2.23	1.24	2.00	*n*	36	25	18	11	6
				%	37.50	26.04	18.75	11.46	6.25
skin sensations, e.g. itching, “pins and needles”, numbness, tingling	2.21	1.16	2.00	*n*	35	25	19	15	2
				%	36.46	26.04	19.79	15.63	2.08
“electrical“ feeling, e.g. similar to an electric shock, like an internal electric shock	1.91	1.20	1.00	*n*	53	15	16	8	4
				%	55.21	15.63	16.67	8.33	4.17
visual stimuli with eyes closed, e.g. flickering, flashes of light	1.88	1.11	1.00	*n*	50	19	20	3	4
				%	52.08	19.79	20.83	3.13	4.17
sudden sitting up, frightened scream, Startling as if in a microsleep	1.74	1.07	1.00	*n*	58	15	15	6	2
				%	60.42	15.63	15.63	6.25	2.08
acoustic stimuli without an external sound source, e.g. loud, explosive noise in the head	1.38	0.82	1.00	*n*	75	11	6	3	1
				%	78.13	11.46	6.25	3.13	1.04

Table D. Frequencies of accompanying emotions/feelings (multiple answers)

How do you feel right now after a sleep start? How often do you experience these feelings immediately after a sleep start?	*M*	*SD*	*Md*		*never (1)*	*sporadically (2)*	*occasionally (3)*	*often (4)*	*very often (5)*
alarmed/alert	3.00	1.37	3.00	*n*	20	15	20	27	14
				%	20.83	15.63	20.83	28.13	14.58
confused/disoriented	2.94	1.36	3.00	*n*	20	18	19	26	13
				%	20.83	18.75	19.79	27.08	13.54
frightened/panicked – danger!	2.80	1.43	3.00	*n*	27	15	17	24	13
				%	28.13	15.63	17.71	25.00	13.54
anxious/worried	2.85	1.48	3.00	*n*	25	20	12	22	17
				%	26.04	20.83	12.50	22.92	17.71
wide awake/clear/fully alert	2.54	1.20	2.00	*n*	21	30	24	14	7
				%	21.88	31.25	25.00	14.58	7.29
neutral	2.47	1.23	2.00	*n*	23	33	21	10	9
				%	23.96	34.38	21.88	10.42	9.38
desperate/sad/depressed	2.14	1.30	2.00	*n*	44	19	16	10	7
				%	45.83	19.79	16.67	10.42	7.29

Table E. Frequencies of short-term consequences (multiple answers).

What happens immediately after a sleep start?	*M*	*SD*	*Md*		*never (1)*	*sporadically (2)*	*occasionally (3)*	*often (4)*	*very often (5)*
I usually fall asleep straight away	3.34	1.09	4.00	*n*	4	21	22	36	13
				%	4.17	21.88	22.92	37.50	13.54
Then I try to force myself to fall asleep	2.75	1.33	3.00	*n*	23	19	24	19	11
				%	23.96	19.79	25.00	19.79	11.46
I'm wide awake, for now, falling asleep is no longer possible.	2.36	1.10	2.00	*n*	23	37	16	18	2
				%	23.96	38.54	16.67	18.75	2.08
Afraid of falling asleep again, because it will probably happen again.	1.84	1.24	1.00	*n*	58	13	13	6	6
				%	60.42	13.54	13.54	6.25	6.25
Afraid of the next day	1.81	1.11	1.00	*n*	54	17	18	3	4
				%	56.25	17.71	18.75	3.13	4.17
Afraid of health causes	1.74	1.18	1.00	*n*	62	13	9	8	4
				%	64.58	13.54	9.38	8.33	4.17
Afraid of health consequences	1.52	1.08	1.00	*n*	72	11	4	5	4
				%	75.00	11.46	4.17	5.21	4.17

Table F. Frequencies of accompanying cognitions (multiple answers).

Are there certain thoughts that keep coming back or just flashing when you fall asleep, shortly before or after you wake up?	*M*	*SD*	*Md*		*never (1)*	*sporadically (2)*	*occasionally (3)*	*often (4)*	*very often (5)*
"I finally have to sleep!"	3.15	1.32	3.00	*n*	16	13	24	27	16
				%	16.67	13.54	25.00	28.13	16.67
I'm thinking of specific private concerns	2.78	1.28	3.00	*n*	19	24	22	21	10
				%	19.79	25.00	22.92	21.88	10.42
I think about (non-stressful) everyday things	2.63	1.27	3.00	*n*	24	22	23	20	7
				%	25.00	22.92	23.96	20.83	7.29
I'm thinking of specific professional concerns	2.27	1.13	2.00	*n*	30	28	23	12	3
				%	31.25	29.17	23.96	12.50	3.13
"The time has come, I'll fall asleep any minute!"	1.96	1.27	1.00	*n*	54	12	15	10	5
				%	56.25	12.50	15.63	10.42	5.21
"I must not fall asleep!" / "Danger!"	1.39	0.81	1.00	*n*	73	14	5	3	1
				%	76.04	14.58	5.21	3.13	1.04

G. Open-text responses to the “Other” option for individual symptom descriptions and associated experiences.

Legend:
a) Individual Symptom Descriptions
b) Co-occurring Symptoms
c) Associated Emotions
d) Associated Cognitions
Grey font: answers provided by participants in the comparison group (FoSS < 1)

- **a)** Most of the time, it is the feeling of suddenly falling very deeply, and then my body jerks and I am fully awake again.
  b) The muscle jerk and the feeling of falling.
- **a)** Very sudden and sometimes somewhat strange.
  b) No.
- **a)** I dream that I stumble or fall, and to keep my balance I suddenly take a step in the dream. In reality, however, my leg only jerks or kicks, and I wake up because of that movement. This always happens while I am falling asleep, and then it takes a while before I can fall asleep again because of the adrenaline.
- **a)** It feels terrible; you think there is something physically wrong with your body, and it is difficult to get back into the process of falling asleep afterwards.
  b) No, not at the same time, but alternating.
- **a)** It feels as if I am falling through the mattress or dropping down onto the mattress from above; then I startle awake and am fully alert again.
  b) Dreams already occurring at the moment of falling asleep, together with the sensation of falling and then suddenly sitting upright.
- **a)** It feels as if I am falling from somewhere.
  b) Heart pounding and muscle jerks.
- **a)** Sometimes I startle awake when I am dreaming something. Once I am awake, I realize that the dream was not real.
- **a)** Sometimes startling; mostly annoying/exhausting because I have woken up.
  b) Leg jerks triggered by dream experiences.
  c) Annoyed.
- **a)** As if my chest were being squeezed and I couldn't get any air.
  b) No
- **a)** I think that I am falling from somewhere or that someone is startling me.
  b) Yes, the jerks and the scream of fear, so more the startle itself. If something bad happens in the dream, I immediately startle awake.
- **b)** The feeling of falling and then being startled.
- **a)** A sudden startle accompanied by a jerk in one part of the body, and afterwards I am briefly very awake again.
  b) The startle feels like the main reason; the jerks occur alongside it (but I'm not entirely sure). My girlfriend often notices me twitching shortly before I fall asleep because she can tell I'm drifting off.
- **a)** One body part (leg, arm) suddenly jerks, like a muscle twitch or an electric jolt. Sometimes it also feels like being startled awake from a kind of micro-sleep.
  b) Muscle twitching, electric sensations, being startled awake from a split-second sleep.
  c) Frustrated and annoyed because I wanted to fall asleep and cannot explain or prevent this phenomenon.
- **a)** It doesn't happen often, but when it does, I either jerk very strongly and wake up because of it, OR for a brief moment it feels as if I am falling. Both are unpleasant, and I am glad it does not happen more often.
  b) No, usually they occur separately. Sometimes I also have a racing heartbeat for a few seconds after the sensation of falling.
- **a)** Usually I am falling in a dream and startle awake at or because of the impact.
- **a)** It feels as if I am falling, and I startle very strongly because I want to prevent it. I then wake up and realize that I am holding onto the bed and that my heart is beating faster. Afterwards there is great relief when I realize that I am simply lying normally in bed.
  b) Yes, racing heart, dizziness, and deep relief once it is over.
- **a)** I find it very unpleasant. If I startle awake while falling asleep, it usually takes me longer to calm down again.
  b) No
- **a)** Brief shock. Brief disorientation. Realizing that I had apparently been holding my breath. Then everything is fine again.
  b) Muscle twitching, sensation of falling.
- **a)** The startle itself is nothing special. I simply wake up.
  b) No
- **a)** It is a shock; you feel groggy and first have to calm down and figure out where you are and what just happened.
- **a)** Stress, frustration/anger because falling asleep is often so difficult; sometimes fear because the sensation that woke me up was very unpleasant.
  b) Heart pounding and shortness of breath.
  c) angry
- **c)** I watch videos on YouTube because I know I won't be able to fall asleep again without some kind of calming external stimulus. Sometimes I also eat something because I occasionally wake up hungry.
- **c)** Usually shortly after falling asleep. Often I am jumping off a cliff into the depths and wake up because of it. I am certain it is triggered by stress (exam stress, relationship problems, family problems). When these stressors are present, I have nightmares almost every night.
  b) Only the sensation of falling and the sudden startle awake, and sometimes additionally sitting upright.
- **a)** During the process of falling asleep, like in a dream—for example, I am walking and then trip or fall, and then I jerk awake.
- **a)** Usually I dream that I am falling or losing the ground beneath my feet, and when my leg physically steps into empty space, I wake up with a jolt.
  b) After certain dreams I wake up abruptly, with an increased pulse and sweating. During some dreams I start talking and moving, and sometimes I jerk very strongly and wake up at the same time, usually accompanied by an unsettling feeling and occasionally an elevated pulse.
  c) angry
- **a)** Becoming awake again very suddenly; afterwards usually confused and exhausted.
- **a)** Fear, discomfort, unease.
  b) Racing heart and sitting upright; racing heart and falling sensations.
- **a)** Most often the startle occurs during the process of falling asleep. Very rarely I have a brief muscle twitch (perhaps twice a year), and then I startle awake. Otherwise it is more of a mental startle because a good idea comes to me while half asleep and I want to write it down.
  b) No
- **a)** Very confusing; it happens very suddenly and startles me.
- **a)** Usually I suddenly notice that my whole body jerks, which wakes me up again. Often I only then realize that I had already started dreaming. At other times I know that I am dreaming but cannot move my body, and only after prolonged efforts to wake up do I finally manage it.
  b) A loud noise (bang/explosion) and muscle jerks. Dreams during sleep onset and muscle jerks. For me, being startled and muscle jerks are basically the same thing.
  c) I think about what I was just dreaming/experiencing. Sometimes I

briefly try to sort out what belonged to the dream and what belonged to reality.

- **a)** Sometimes the startle is caused by muscle jerks, or often by the sensation of falling into the depths; afterwards I have heart palpitations and feel agitated.
  b) Rarely or never.
- **a)** Often there is a loud "bang" or I see flickering images before my eyes.
- **a)** As if I were falling. It is very sudden and very quick and usually happens when I am almost asleep.
  b) No
- **a)** Usually I am already dreaming and in the dream I slip or fall down a staircase. This makes me jerk, and I wake up from the jerking.
  c) Amused
- **a)** Like a shock moment in which you are startled. Complete calm beforehand followed by a sudden fright. Comparable to a car accident you were not expecting (for example, being rear-ended while stopped at a red light).
  b) The sensation of falling and muscle jerks occur together more often than separately.
- **a)** Frightening. I wake up and realize that I cannot move. I want to scream or turn on the light, but I cannot. I try to move, but it doesn't work.
  b) Muscle jerks and the sensation of falling. Being unable to move and an elevated pulse; it feels like a panic attack.
- **a)** Intense fear. I usually hear a scream, a loud humming noise, or a "catastrophe sound," sometimes even my name. I am generally more fearful in the dark, and afterwards even more so. I often feel "crazy."
  b) Usually followed by faster heartbeat and faster breathing.
- **a)** In my dream I feel/see myself falling, and then I am suddenly awake again.
  b) Muscle jerks and waking up.
- **a)** My legs or arms jerk, and I wake myself up because of it.
  b) If I have not moved much during the day, my legs often tingle unpleasantly when I go to bed.
  c) Tired and annoyed that I cannot sleep.
  d) I calm myself down and meditate a little / focus on my breathing (also to distract myself from the tingling in my legs).
- **a)** Unexpected; heart pounding;
  not sure where I am or what day/time it is.
- **a)** Like falling; adrenaline shoots through my whole body.
- **a)** The startle frightens and stresses me; I get a racing heart. Afterwards it is difficult to fall asleep again.
  b) Hearing a loud noise and simultaneously jerking/shuddering as if from an electric shock.
  c) Finally I can relax and let go.
- **a)** When I am falling asleep, it often feels as if I am falling and my breathing and heartbeat slow down (which is normal), but because of an irrational fear that they might become TOO slow, I often startle awake at that moment before I am actually asleep.
  b) Dreams before falling asleep and the sensation of falling.
  c) For example, I try to recite rhymes or songs to myself, imagine fantasy worlds, etc., in order to consciously distract myself and help myself fall asleep.
- **a)** Most of the time it happens within a kind of "half-dream." I am walking down stairs or flying and then falling. That wakes me up and startles me briefly. Sometimes it also happens without dreaming and I simply startle awake. I imagine it looks like someone being resuscitated, and especially in the beginning my husband often asked what was wrong. By now it is just part of life.
  b) Startling awake and dreaming; racing heart or a strong feeling of fear only when I wake up from deep sleep or REM sleep and had actually already been dreaming, and the dream felt very realistic.
- **a)** Startling; rapid heartbeat.
  b) Occur together.
- **a)** I rarely know anything about my nighttime activities. My partner points them out to me.
  c) No memory.
- **a)** As if I were falling into a deep hole or being pulled downward. The sensation of falling sometimes feels like a dream while half asleep, but not always. Recently I have generally had fewer such experiences since moving my sleeping place to a quieter environment (without street noise, partner's snoring, etc.).
- **a)** Startling awake happens very rarely for me. When it does, it is usually because of a dream. In that moment I feel confused and slightly agitated.
- **a)** I briefly jerk as if I were cold. Since I know the feeling, I do not think much about it. Sometimes I get annoyed because I would like to be asleep already.
  b) No
  c) angry
- **a)** Sometimes I simply jerk and nothing else happens, and I fall asleep again quickly; sometimes I suddenly feel completely awake and have a racing heart, which is very unsettling and makes it take a while before I can sleep again.
  b) Racing heart and startling awake/sitting upright.
- **a)** Occasionally I am already dreaming while falling asleep and then I fall in the dream and get startled. More often, however, during sleep onset I experience muscle jerks in my legs or itching that spreads from my head across my whole body without any external trigger. It is very unpleasant. If heart pounding or a racing heart is added to this, I find it very difficult to fall asleep.
  b) Yes, itching and heart pounding.
- **a)** Racing heart; I see figures/outlines in the room, which disappear only after blinking several times. Sometimes I also see black dots (like flickering) or a spider web above my bed. The outlines are not always black; sometimes they are white.
  b) Racing heart every time, followed by difficulty falling asleep. Otherwise as described above.
- **a)** In my own apartment I usually know exactly where I am and where the noise came from. In other places I feel disoriented and briefly very confused until I can place my surroundings again. When I have a muscle jerk, I briefly startle because it is sometimes linked to a dream. Then I realize that I was in the process of falling asleep and let myself drift back into sleep.
  b) Muscle jerks and dreams during sleep onset occur together. Noises that wake me up and suddenly sitting upright also occur together.
  c) I feel as if I am in another world; I am still in the dream world.
  d) I am falling asleep. That is good.
- **a)** When I startle awake, I have an elevated pulse, racing heart, a feeling of fear, and brief disorientation until I find a point of reference in my surroundings.
  b) No
  c) If the cause was external, I cannot fall asleep again immediately.
- **a)** Brief fright, eyes wide open, taking a deep breath, sitting up slightly, and in the same moment realizing that everything is fine.
  b) Jumping awake and opening my eyes wide.
- **a)** Usually only a mild startle. I have the feeling that some thought in my head triggers the awakening, but afterwards I can no longer grasp it. Abruptly becoming alert; afterwards feeling awake and unable to fall asleep again → mild frustration.
  b) Sensation of falling and muscle jerks; dreams and muscle jerks; dreams and accelerated heartbeat.
  c) Frustrated because I want to use my sleeping time and know that once I have startled awake, I will not fall asleep again easily.
- **a)** I am afraid of suffocating and then have to cough.
- **a)** It hardly ever happens. Not really startling awake, just waking up.
  b) No
- **a)** Loss of control. I have fallen asleep, but then somehow not (that is what it feels like). The sensation that I stumble or fall.
  b) No, only one of those things.
- **a)** For me, the startle is like a warning signal, as if I suddenly found myself in a burning house. Instantly I am wide awake, alert, and paying attention to every tiny detail. I react before I think.
  b) No, at least I cannot remember.
- **a)** Sensation of falling during sleep onset; startled, but I can calm down again easily.

- **a)** Sometimes I do not remember it, but my partner tells me about it. When I do notice it, it is often linked to the perceptions mentioned above, such as seeing something in the room. It may be a man, someone trying to attack me, insects, or other perceptions. When I startle awake, I usually lie back down and continue sleeping. If someone asks me what happened, I sometimes become aggressive because on some level I realize that what I saw does not make sense.
  b) Startling awake sometimes several times a week.
- **a)** Most of the time, the startle is accompanied by muscle jerks and the feeling that I have "jumped up" or "sprung up." I usually feel very startled and frightened. For a short time I am fully awake, but afterwards I calm down quickly. Usually accompanied by a racing heart and a little panic, which quickly passes. Most of the time I feel that external stimuli triggered the startle, even when it is completely quiet.
  b) Muscle jerks, panic, racing heart, fright, and the sensation of "jumping up" usually occur together.
- **a)** As if I had stepped down a stair.
- **a)** I fall asleep very quickly, within three minutes (sometimes even faster when I am extremely tired), and very often I start dreaming almost immediately, or my thoughts blend into bizarre dreams. In them I often miss a pothole or the last step of a staircase. Sometimes I also talk in my dream, which comes out as a strange sound in real life and wakes me up.
  b) No
- **a)** I experience it first as a "shock", often disoriented and unable to identify it immediately.
- **a)** Restlessness.
- **a)** A jolt running through my entire body that wakes me up again.
  b) No, I do not think so.
  c) Annoyance when it happens several times during sleep onset.
- **a)** A twitch in my chest. You think you cannot breathe even though everything is actually fine.
  b) Twitching and the sensation of shortness of breath always occur together.
- **a)** Not really worried; rather amused when it happens occasionally. Relieved that I was only dreaming or that I had not overslept.
  b) no
  c) Amused and relieved.
- **a)** Not good, exhausting.
- **a)** Loud noises bother me during the process of falling asleep.
  b) no
- **a)** It feels like being startled.
  b) Sensation of falling, muscle jerks, and elevated pulse.
- **a)** I am startled and initially disoriented. Sometimes I am annoyed that I woke up again. If it concerns unfinished tasks, those thoughts often keep me from falling asleep again for quite a while.
  b) Not that I know of.
- **a)** Stress, disorientation, hecticness.
  b) All together.
- **a)** Annoying. The more often you wake up, the less sleep time remains.
  b) Tired the next day.
- **a)** I am tired and worried about what happened. I really do not want to worry; I just want to sleep.
  b) Annoyed because nothing actually happened.
- **a)** A feeling of disorientation and confusion.
  b) Yes, visual stimuli with closed eyes, e.g., flickering, flashes of light / heart pounding, elevated pulse, rapid breathing / other sensations such as feeling like I am falling or fainting.
- **a)** A brief simultaneous jerk through all limbs.
- **a)** Falling from a ladder / plunging into a hole (visual experience, slight dizziness), feels like being "pulled."
- **a)** Since in some periods I have severe difficulties falling asleep on two out of three nights (lying awake in bed for 2–3 hours), I feel relieved once I finally fall asleep. If something then wakes me up again, it first feels like a brief shock, similar to a skipped heartbeat, and afterwards mainly irritation that I am awake again.
  b) Usually sudden tingling and itching in several parts of my body initially prevent me from falling asleep, and often a second time because of a jumping leg or arm.
  c) As already mentioned: annoyed that I am awake again.
  d) That's it—I won't be able to fall asleep again tonight.
- **a)** When I wake up because of my partner's noise (an explosive, loud exhalation), I jump awake and am initially very aggressive and angry. It is very unpleasant; it feels as though I have been put on high alert. I think the rising anger is also because it is already so difficult for me to get into sleep mode in the first place, and afterwards falling asleep is often impossible again for a long time. When I wake myself up (more rarely) because of a sudden jerk, it lasts about a second; I quickly regain my bearings and can usually return to the process of falling asleep.
- **a)** Not very bad.
  b) No
- **a)** A sudden jerk. I immediately know that I was just falling asleep. What feels like 1–2 minutes later, I am asleep again.
- **a)** Usually like an electric shock or the sensation of falling.
  b) no
- **a)** Mostly annoyed.
  b) Yes, excessive sensitivity to heat and noise.
- **a)** Sudden sensation of falling.
  b) No
- **a)** Occasionally, within the first hour after going to bed, I wake up because of one of the phenomena mentioned above and then feel awake, as if I had taken a power nap. I do NOT experience this awakening as a startle response, but rather as a surprising and unwanted interruption of sleep.
  b) No, the symptoms usually occur separately.
- **a)** I am very irritated by it; at first I do not know who I am or where I am. Sometimes I am also afraid and have the feeling that a stranger or some greater power is in the room.
  b) They rarely occur together.
- **a)** It is almost exactly the same as the shock you get when you "step into empty space." For example, when you miss a curb and step from the sidewalk toward the street, or when you are walking up stairs in the dark, expect another step, but are already at the top and unnecessarily lift your leg too high. This shock always affects only one leg. You wake up briefly, realize that you are lying safely in bed, and immediately fall asleep again. I have not experienced this effect for many years. It tended to occur when I was very tired and fell asleep quickly. Always shortly after falling asleep (estimated maximum of 30 minutes). Often accompanied by a dream, although the dream content was not the trigger (as far as I can remember, which has become increasingly difficult with age). It may have been more common during stressful periods of life. Stress in the sense of unresolved issues, not excessive workload.
  b) No, only muscle jerks. More like a single kick, actually. Not a prolonged "fluttering" twitch.
- **a)** During a period of extreme stress (moving house), I experienced "muscle jerks" again for the first time in years and was unable to fall asleep because of them (a completely sleepless night). As a child, I often had the sensation of startling awake as if from a microsleep, but that was years ago. As a child it was accompanied by thoughts such as "It's about to happen, I'm falling asleep," or "I have to fall asleep now" (see follow-up question).
- **a)** A) Taking a deep breath accompanied by a startled feeling in my chest. B) Being dragged reluctantly out of sleep with sleep paralysis.
  b) Don't know.
  c) Circular thoughts. My mind just keeps thinking. "Do penguins actually have knees?"
  d) I cannot sleep again for hours and listen to an audiobook.
- **a)** I often fall asleep with negative thoughts, worries, or sadness. After suddenly waking up, these thoughts immediately return. In that moment I wish I could have just kept sleeping and escaped those thoughts and feelings. Falling asleep again usually takes even longer than the initial process of falling asleep. I am aware of this in the moment, which makes it even more distressing.
  b) Being very tired but unable to continue sleeping, preoccupied with thoughts.

- **a)** Annoying, disappointing, exhausting.
  b) All together.
- **a)** It varies; there is no pattern. Usually an intense feeling of fear, as if I had been abruptly pulled out of something.
  b) They do not usually occur together.
- **a)** Startle, fear, confusion, partial disorientation, fright, shock, loneliness, sadness, panic.
  b) All in different combinations; most commonly fright, shock, panic, confusion, and fear.
  c) What is happening? What is this? (Sometimes I try to hold on to images that flash before my eyes for a fraction of a second.) Everything is okay, I am in my bed.
  a) Most of the time the dream feels real.
  b) Sometimes.
- **a)** Sometimes the sensation of falling; often my arms or legs fall asleep; I have a lot of joint, muscle, and hip pain; sometimes many thoughts in my head... I am tired but cannot quite get past the "point" of falling asleep; sometimes noises disturb me, e.g., snoring.
- **a)** 1. The sensation of falling and waking up at the exact moment of impact—the dream itself is not remembered afterwards, but there is a definite feeling of having dreamed. 2. After searching for a comfortable sleeping position, feeling my body relax at the moment of falling asleep, then losing the position and experiencing a physical discomfort.
  b) no
  c) Relieved.
- **a)** I get startled
  b) No
- **a)** Unfounded fear of a threat or a fall.
  b) A scream and an elevated pulse.
- **a)** Especially when several stressful situations occur at once, I wake up from the slightest twitch (often even a finger moving briefly is enough) or from a swallowing reflex; sometimes there is flickering light in my vision, rarely a noise in my head, and sometimes the sensation of stepping off into empty space.
  b) Most of the time these are muscle jerks—not directly simultaneous, but occurring one after another during sleep onset, e.g., a twitch followed by a swallowing reflex.
- **a)** The sensation of suddenly falling, usually without dreaming anything related to it. Sometimes just a sudden "fright." Overall it has become very rare in recent years.
- **a)** As if there were a loud bang or something similar.
  b) no
- **a)** Frightening, brief sequence of several bizarre events; confusion upon waking; sometimes accompanied by tears, extreme anxiety, heart pounding, shortness of breath, dry throat; I feel drained and restless.
  b) Tears, anxiety, dry throat, heart pounding.
- **a)** I get startled.
  b) No
- **a)** Disorientation, concern, oppression.
  b) Concern and oppression.
  c) Restlessness, followed by insomnia.
  d) I get up and stay awake.
- **a)** Either because of an external noise or, more often, because of a dream suggesting that an unknown person (99% of the time male) is in the room. I feel threatened or frightened.
  b) Threat and fear.
- **a)** I immediately recognize that it is one of those sleep-onset jerks, turn over, and try to continue falling asleep.
  b) Don't know.

Table H. Conditions subjectively influencing the FoSS (multiple answers).

Do the Sleep starts become more or less frequent under certain conditions?	*M*	*SD*	*Md*		*much less often (1)*	*less often (2)*	*unchanged (3)*	*more often (4)*	*much more often (5)*
when I am not feeling well mentally	3.70	0.80	4.00	*n*	1	3	34	44	14
				%	1.04	3.13	35.42	45.83	14.58
when I am tense / stressed	3.64	0.82	4.00	*n*	2	3	35	44	12
				%	2.08	3.13	36.46	45.83	12.50
private concerns	3.61	0.72	4.00	*n*	1	2	38	47	8
				%	1.04	2.08	49.58	48.96	8.33
occupational concerns	3.38	0.68	3.00	*n*	1	4	53	34	4
				%	1.04	4.17	55.21	35.42	4.17
in unusual sleeping conditions (e.g. not at home, bed partner present/absent)	3.18	1.04	3.00	*n*	8	10	45	23	10
				%	8.33	10.42	46.88	23.96	10.42
if I am not feeling well physically	3.13	0.77	3.00	*n*	3	10	59	20	4
				%	3.13	10.42	61.46	20.83	4.17
in case of sleep deprivation / after a bad night	3.07	0.94	3.00	*n*	7	11	53	18	7
				%	7.29	11.46	55.21	18.75	7.29
if I often fight against microsleeps / involuntary nodding off during the day	2.79	0.82	3.00	*n*	11	10	64	10	1
				%	11.46	10.42	66.67	10.42	1.04
when I am balanced / relaxed	2.19	0.85	2.00	*n*	24	33	36	3	0
				%	25.00	34.38	37.50	3.13	0.00

Table I. Countermeasures (multiple answers)

Have you attempted any of the following methods to reduce sleep starts? If so, how often?	*M*	*SD*	*Md*		*never (1)*	*sporadically (2)*	*occasionally (3)*	*often (4)*	*very often (5)*
tried to think about something else	3.14	1.44	3.00	*n*	21	10	20	25	20
				%	21.88	10.42	20.83	26.04	20.83
reduced external sources of disturbance	2.53	1.52	2.00	*n*	39	11	16	16	14
				%	40.63	11.46	16.67	16.67	14.58
exercise	2.29	1.26	2.00	*n*	37	17	24	13	5
				%	38.54	17.71	25.00	13.54	5.21
relaxation exercises	2.24	1.28	2.00	*n*	42	10	29	9	6
				%	43.75	10.42	30.21	9.38	6.25
sleeping pills / sedatives	1.43	0.96	1.00	*n*	75	10	5	3	3
				%	78.13	10.42	5.21	3.13	3.13
psychotherapy	1.36	0.92	1.00	*n*	81	3	5	6	1
				%	84.38	3.13	5.21	6.25	1.04
alcohol to fall asleep	1.30	0.65	1.00	*n*	75	15	4	2	0
				%	78.13	15.63	4.17	2.08	0.00

Table J. Efficacy of countermeasures (multiple answers).

If you have tried any of these: Has the frequency of sleep starts changed?	*M*	*SD*	*Md*		*much less often (1)*	*less often (2)*	*unchanged (3)*	*more often (4)*	*much more often (5)*	*not tried*
psychotherapy	2.67	0.62	3	*n*	1	3	11	0	0	81
				%	1.04	3.13	11.46	0.00	0.00	84.38
tried to think about something else	2.63	0.91	3	*n*	9	16	36	5	2	28
				%	9.38	16.67	37.50	5.21	2.08	29.17
reduced external sources of disturbance	2.59	0.86	3	*n*	6	12	29	0	2	47
				%	6.25	12.50	30.21	0.00	2.08	48.96
Exercise	2.61	0.94	3	*n*	7	19	29	3	3	35
				%	7.29	19.79	30.21	3.13	3.13	36.46
Relaxation exercises	2.60	0.88	3	*n*	6	18	28	3	2	39
				%	6.25	18.75	29.17	3.13	2.08	40.63
Alcohol to fall asleep	2.42	0.90	3	*n*	4	9	12	0	1	70
				%	4.17	9.38	12.50	0.00	1.04	72.92
Sleeping pills / sedatives	2.09	1.06	2	*n*	8	6	7	0	1	74
				%	8.33	6.25	7.29	0.00	1.04	77.08

Note: Descriptive statistics were calculated using only participants who had tried the respective strategy; responses in the "not tried" category were excluded from the analyses.

Table K. Pre-existing physical illnesses, mental disorders, and substance use

Diagnosis	*SS group (FoSS ≥1)*	*comparison group (FoSS <1)*	*χ^2-test*
**Physical illness (yes)**	28	19	$\chi^2(1) = 2.53$, $p = .112$
Hypertension	1	5	$\chi^2(1) = 2.67$, $p = .212$°
Thyroid disease	9	8	χ^2 (1) = 0.09, $p = .765$
Chronic pain	4	4	χ^2 (1) = .001, $p = .976$
Vertigo	4	1	χ^2 (1) = 1.91, $p = .209$°
**Mental disorders (yes)**	25	14	$\chi^2(1) = 4.17$, $p = .041$, Cramers $V = .147$
Anxiety	6	7	$\chi^2(1) = 0.11$, $p = .745$
Depression	9	8	$\chi^2(1) = 0.09$, $p = .765$
Sleep disorders	14	2	$\chi^2(1) = 10.08$, $p = .001$, Cramers $V = .228$
Nightmares	10	1	$\chi^2(1) = 8.01$, $p = .005$, Cramers $V = .203$
PTBS	3	0	$\chi^2(1) = 3.11$, $p = .119$°
**Substance use**	**never:**		never / occasionally/ often/daily
Caffeine	13	11	$\chi^2(3) = 3.72$, $p = .293$
Alcohol	13	18	$\chi^2(3) = 2.71$, $p = .438$
Nicotine	69	77	$\chi^2(3) = 2.18$, $p = .537$
Sedativa	83	91	$\chi^2(3) = 3.88$, $p = .275$
Antidepressants	91	94	$\chi^2(2) = 1.03$, $p = .598$
Stimulants	84	94	$\chi^2(3) = 5.47$, $p = .141$

°Expected frequencies < 5: Fishers exact test

Table L. Personal and occupational concerns

Diagnosis	*SS group (FoSS ≥1)*	*comparison group (FoSS <1)*	*χ^2-test*
**Personal concerns (yes)**	46	28	$\chi^2(1) = 7.69$, $p = .006$, Cramers $V = .199$
**Occupational concerns (yes)**	34	29	$\chi^2(1) = 0.75$, $p = .386$

Table M. Sensitivity analysis, 25th percentile

Variable	*Groups: distribution measures* SS (n = 109, FoSS ≠ 0.00)			Non-SS (n = 85, FoSS = 0.00)			*Correlation (df = 107)* Variable, FoSS		Mann–Whitney U test SS vs. non-SS		
	M	*SD*	*Md*	*M*	*SD*	*Md*	r_s	*p*	*Z*	*p*	*r*
Age (years)	30.50	10.99	28.00	35.73	15.43	31.00	–.05	.657	–2.10	.047*	.15
PSQI total	6.94	3.55	6.00	5.22	2.35	5.00	.34	.002**	–3.42	.004**	.25
SSQ	1.35	0.70	1.00	0.91	0.57	1.00	.33	.002**	–4.42	.004**	.32
efficiency %	83.54	11.90	85.71	87.92	11.11	88.89	–.13	.250	–2.85	.009**	.21
SOL (min)	33.52	28.08	30.00	24.81	25.71	15.00	.13	.250	–2.52	.017*	.18
TST (h)	6.80	1.15	7.00	7.04	1.16	7.00	–.10	.358	–1.60	.129	.12
ISI	10.27	5.99	11.00	5.28	3.85	5.00	.37	.002**	–5.99	.004**	.43
ESS	7.81	3.81	7.00	7.04	3.87	7.00	.01	.935	–1.37	.172	
MZS	63.06	26.23	60.00	52.15	20.88	49.00	.37	.002**	–3.06	007**	.22
HADS anxiety	7.39	3.90	7.00	5.72	3.13	5.00	.34	.002**	–2.89	.009**	.21
HADS depression	4.83	4.39	4.00	3.65	3.32	3.00	.24	.022*	–1.52	.140	
HADS total	12.22	7.38	10.00	9.36	5.65	8.00	.32	.002**	–2.60	.015*	.19
Importance of sleep	2.17	0.69	2.00	1.89	0.69	2.00	.29	.004**	–2.65	.015*	.19

p-values for correlations and Mann–Whitney U tests were adjusted for multiple comparisons using the Benjamini–Hochberg procedure.
* $p < .05$. ** $p < .01$. *** $p < .001$

Table N. Sensitivity analysis, 75th percentile

Variable	*Groups: distribution measures* SS (n = 55, FoSS ≥ 2.00)			Non-SS (n = 139, FoSS < 2.00)			*Correlation (df = 53)* Variable, FoSS		Mann–Whitney U test SS vs. non-SS		
	M	*SD*	*Md*	*M*	*SD*	*Md*	r_s	*p*	*Z*	*p*	*r*
Age (years)	29.95	10.99	26.00	33.91	14.04	29.00	.17	.220	–1.77	.091	.13
PSQI total	7.91	3.66	8.00	5.51	2.71	5.00	.28	.039*	–4.58	.002**	.33
SSQ	1.56	0.76	2.00	0.99	0.57	1.00	.20	.383	–5.13	.002**	.37
efficiency %	82.43	12.43	85.71	86.66	11.27	87.50	–.20	.145	–2.24	.032*	.16
SOL (min)	36.80	27.60	30.00	26.90	26.83	15.00	–.02	.913	–2.76	.010*	.20
TST (h)	6.73	1.24	7.00	6.98	1.12	7.00	–.09	.497	–1.46	.157	.11
ISI	12.45	6.19	12.00	6.35	4.49	6.00	.09	.499	–6.25	.002**	.45
ESS	7.58	3.15	7.00	7.42	4.10	7.00	.16	.237	–0.53	.597	
MZS	71.78	24.32	68.00	52.94	22.62	49.00	.33	.015*	–4.67	.002**	.34
HADS anxiety	8.71	3.91	8.00	5.85	3.24	5.00	.03	.859	–4.60	.002**	.33
HADS depression	5.71	4.83	5.00	3.76	3.47	3.00	.08	.545	–2.56	.015*	.18
HADS total	14.42	7.86	13.00	9.60	5.84	8.00	.04	.786	–4.06	.002**	.29
Importance of sleep	2.33	0.64	2.00	1.94	0.70	2.00	.41	.002**	–3.46	.002**	.25

p-values for correlations and Mann–Whitney U tests were adjusted for multiple comparisons using the Benjamini–Hochberg procedure.
* $p < .05$. ** $p < .01$. *** $p < .001$

O. Instrument: Online-survey

Startling Awake During the Transition to Sleep

Dear Participant,
Thank you very much for taking the time to participate in this study!

General Information about the Study
Many people are familiar with this phenomenon: you are lying in bed, just about to fall asleep — and suddenly you startle awake again.
This study examines what exactly happens during these sudden awakenings and how they affect daily life and sleep quality.
Anyone can participate, regardless of whether or not they have experienced this phenomenon.
The study is part of a research project investigating sleep problems among shift workers and aims to improve their treatment.
Voluntary Participation
Your participation in this study is entirely voluntary. You may withdraw your consent to participate at any time, including at a later date, without providing any reason. Withdrawing from the study will not result in any disadvantages for you.
Withdrawal from the Study
You may discontinue the study at any time and without giving a reason. To do so, click on "Exit survey and delete responses." Your data will then be deleted automatically, and your responses cannot be used.
Risks and Inconveniences
Completing this online questionnaire should not involve any risks or inconveniences for you.
However, if you experience any unexpected distress as a result of participating, please contact the study coordinator (see contact information below).
Confidentiality of Data
Your data will be treated confidentially. All data collected as part of this study will be anonymized using a code before being analyzed. The anonymized research data may be shared with other researchers.
Contact Person / Study Coordinator
If you have any questions during or after the study, please feel free to contact the study coordinator at any time:
Tanja Grünberger, MSc
Department of Psychology, University of Salzburg
Hellbrunner Straße 34, 5020 Salzburg, Austria
tanja.gruenberger@stud.plus.ac.at
Consent Declaration
By clicking "Continue," you confirm that you have read and understood the information above and agree to participate in this survey voluntarily.

General Information
First, we would like to ask you a few questions about yourself and your current life situation.
All information you provide will be analyzed anonymously. It will not be possible to identify you personally.

Gender:

- Female
- Male
- Diverse / Non-binary

Age: _____ years
Marital Status:

- Married / In a registered partnership
- In a relationship
- Single
- Divorced / Separated
- Widowed

What is your current living situation? I live in an apartment / house ...

- Alone
- With my partner
- With child(ren)
- With other family members
- With other people

Sleep Situation: I usually sleep ...

- Alone
- With my partner in the same bedroom
- In separate bedrooms
- In separate homes
- With child(ren) in the bedroom
- With pet(s) in the bedroom

Are there any external factors that regularly interfere with your sleep?

- No, none
- Snoring / other disturbances caused by my partner
- Child(ren) / other family members

- Noise (e.g., street noise)
- Lighting (e.g., too bright)
- Other: _____________________

Which country do you currently live in?

- Austria
- Germany
- Switzerland
- Other country: __________________________

Which federal state / canton do you currently live in? ____

How good are your German language skills?

- Native speaker
- Good / very good
- Good enough for this survey
- I only partly understand the questions or do not understand them at all

Do you suffer from any physical complaints or medical conditions?

- No, none known
- Restless Legs Syndrome
- Sleep apnea syndrome
- Periodic limb movements
- High blood pressure
- Thyroid disorder
- Chronic pain
- Dizziness / vertigo
- For women: menopause
- Other: _____________________

Do you suffer from a mental health condition or disorder?

- No, none known
- Anxiety disorder
- Depression
- Sleep disorder
- Nightmares
- Other: _____________________

Do you use any of the following substances?
For this question, please select two answers per row: one for frequency and one for reason.

substance	*frequency*				*reason*			
	never	*occasionally*	*frequently*	*daily*	*to fall asleep / calm down*	*to wake up*	*enjoyment / other reasons*	*no*
Caffeine (coffee, cola, energy drinks)								
Alcohol								
Nicotine								
Sleep aids / sedatives								
Antidepressants								
Stimulant medications / substances								

Is there currently something that is causing you significant stress or emotional strain?
(Please use keywords only, e.g., separation, illness, caring for relatives, bereavement)

- Yes, in my private life: _____________________
- Yes, in my professional life: _____________________
- No, I currently do not have any major stressors

What is your highest level of education?

- No school diploma
- Compulsory school diploma
- Intermediate school diploma
- Vocational school
- High school diploma / university entrance qualification

What is your highest professional qualification?

- No vocational training

- Completed apprenticeship / vocational training
- Completed university degree / university of applied sciences degree
- Professional further education / advanced qualification

What is your current job position?

- Unskilled / semi-skilled worker
- Skilled worker / qualified professional
- Supervisory / management position
- Not currently employed

What is your current employment status?
(If you have multiple occupations, please select all that apply.)

- Employed
- Self-employed
- Marginal employment / side job
- In vocational training
- University student
- Seeking employment
- Homemaker
- Not currently employed
- Retired
- On medical leave for more than three months

Weekly working hours
Please enter the number of hours. If you are currently not employed, please enter "0".

- Full-time (work or studies), weekly hours: ___
- Part-time (work or studies), weekly hours: ___
- Marginal employment / side job, weekly hours: ___
- Regular overtime? Average per week: ___

What are your usual working hours?

- Regular daytime working hours
- Shift work: regular rotating shifts
- Shift work: irregular shifts
- Not currently employed

Which of the following shifts do you regularly work?

- None: only regular daytime working hours
- None: not currently employed
- Early shift
- Late shift
- Night shift
- Split shift
- Weekend work
- On-call duty

Which industry best describes your current occupation?
For example: manufacturing, administration, healthcare professions, hospitality / tourism, transport / traffic, security, other services (e.g., cleaning, repair services). If you are currently not employed, please write "not currently employed."____

How would you describe your current occupation?
If you are currently not employed, please answer the following questions based on your current life situation as best as possible.

My work is most of the time	*monotonous*				*varied*
I often feel through my work	*underchallenged*				*overwhelmed*
I have control over my specific work processes (i.e., how / when I complete tasks)	*No influence*				*A great deal of influence*
The physical working conditions (e.g., noise, heat, cold, dust, odors) are for me	*Very stressful*				*optimal*
The social working conditions (colleagues, supervisors, customers) are for me	*Very stressful*				*optimal*

Startling Awake During Sleep Onset

Do you sometimes suddenly startle awake while falling asleep?

- No
- Yes, but only due to external causes (e.g., noise)
- Yes, even without any recognizable external cause

How often do you experience any of the following when startling awake during sleep onset?	*never*	*rarely*	*occasionally*	*often*	*very often*
I only startle awake because of external causes, e.g., noises					
Muscle jerks, e.g., in the legs, arms, or head					
Skin sensations, e.g., itching, "pins and needles," numbness, tingling					
An "electrical" sensation, e.g., shock-like or like an internal electric jolt					
Visual stimuli with closed eyes, e.g., flickering, flashes of light					
Auditory stimuli without an external sound source, e.g., a loud explosive sound inside the head					
Heart pounding, increased pulse, rapid breathing					
Suddenly sitting upright, fearful scream, startling as if during microsleep					
Other sensations, e.g., feeling like falling or fainting					
Dreams already occurring at the moment of falling asleep					
Other: Please describe<br>Please describe in more detail what the startling awake during sleep onset feels like for you personally, or what you experience during it.________________________________					

Do some of these symptoms usually occur together? If yes, which ones? ____________________

How do you feel immediately after startling awake?<br>***How often do you experience the following feelings directly after startling awake?***	*never*	*rarely*	*occasionally*	*often*	*very often*
Fully awake / clear-headed / alert					
Confused / disoriented					
Anxious / worried					
Frightened / panicked – danger!					
Alarmed / hypervigilant					
Desperate / sad / downhearted					
Other: Please describe					
Fully awake / clear-headed / alert					

Are there certain thoughts that repeatedly occur — or briefly flash up — during the sleep onset phase shortly before or after startling awake?

I think about specific work-related stressors					
I think about specific personal stressors					
I think about everyday matters (that are not stressful)					
"It's about to happen — I'm going to fall asleep any moment now!"					
"I must not fall asleep!" or "Danger!"					
"I finally need to get to sleep now!"					
Other: Please describe					

What happens immediately after startling awake?

I usually fall back asleep right away					
I become fully awake and cannot think about falling asleep again for a while					
Fear of falling asleep again because it will probably happen again					
Fear of medical causes					
Fear of health consequences					
Fear of the next day					
I try to force myself to fall asleep					
Other: Please describe					

Does the startling awake affect how long it takes you to actually fall asleep?
The time between going to bed and falling asleep on nights with startling awake is:

- Longer, by approximately _____ minutes
- Shorter, by approximately _____ minutes
- Unchanged

How often does startling awake during sleep onset occur for you on average?

- Nights with startling awake per week: ____
- Number of startling awakenings per night: ____

<u>Do you fear</u> that startling awake could lead to any long-term consequences for you?	*Never*	*Rarely*	*Occasionally*	*Often*	*Very often*
Chronic sleep deprivation / sleep disorder					
Excessive daytime sleepiness / microsleep					
Falling asleep becomes increasingly difficult					
Fear of going to sleep					
Serious illness					
Physical complaints					
Reduced performance / concentration					
Making mistakes / causing accidents					
Job loss					
Relationship problems (e.g., due to irritability or because a partner also wakes up)					
Social withdrawal (e.g., taking it easy in order not to jeopardize sleep)					
Increasing anxiety / worries, persistently elevated tension					
Increasingly depressed mood, despair, helplessness, lack of motivation					
Rumination / repetitive thoughts					

Have one or more of the following consequences <u>actually occurred</u> as a result of startling awake?					
Chronic sleep deprivation / sleep disorder					
Excessive daytime sleepiness / microsleep					
Falling asleep becomes increasingly difficult					
Fear of going to sleep					
Serious illness					
Physical complaints					
Reduced performance / concentration					
Making mistakes / causing accidents					
Job loss					
Relationship problems (e.g., due to irritability or because a partner also wakes up)					
Social withdrawal (e.g., taking it easy in order not to jeopardize sleep)					
Increasing anxiety / worries, persistently elevated tension					
Increasingly depressed mood, despair, helplessness, lack of motivation					
Rumination / repetitive thoughts					

	Not at all	*Slightly*	*Moderately*	*Strongly*	*Very strongly*
The <u>startling awake itself</u> is distressing to me...					
The <u>fear of negative consequences</u> caused by startling awake is distressing to me...					
The <u>actual consequences</u> of startling awake are distressing to me...					

Does the startling awake become more or less frequent under certain conditions?	*Much less frequent*	*Less frequent*	*Unchanged*	*More frequent*	*Much more frequent*
During work-related stress					
During personal stress					
When I feel balanced / relaxed					
When I feel tense / stressed					
During sleep deprivation / after a poor night's sleep					
When I struggle during the day with microsleep / unintended dozing off					
When I do not feel physically well					
When I do not feel mentally well					
Under unusual sleeping conditions (e.g., not sleeping at home, partner present/absent)					

Have you ever tried any of the following methods to reduce the startling awake? If yes, how often?	*Never*	*Rarely*	*Sometimes*	*Often*	*Very often*
Sleep aids / sedatives					
Alcohol to help fall asleep					
Exercise / sports					
Relaxation exercises					
Psychotherapy					
Tried to think about something else while falling asleep					
Reduced external disturbances					
Other, please describe: _______________________					

If you have tried any of these methods:<br>Did they change how often the startling awake occurred?	*Not tried*	*Much less frequent*	*Less frequent*	*Unchanged*	*More frequent*	*Much more frequent*
Sleep aids / sedatives						
Alcohol to help fall asleep						
Exercise / sports						
Relaxation exercises						
Psychotherapy						
Tried to think about something else while falling asleep						
Reduced external disturbances						
Other, please describe: ___________________________						

Sleep Quality (PSQI)

Daytime Sleepiness (ESS)

If you have unintentionally fallen asleep during the day, what thoughts go through your mind when you wake up?

- I never unintentionally fall asleep.
- No thoughts or neutral thoughts, e.g., "I guess I fell asleep."
- Warning thoughts, e.g., "I must not fall asleep!" "Attention! Danger!"
- Self-blame, e.g., "How could you possibly fall asleep in a situation like this?"
- Helplessness, e.g., "There is nothing I can do to stop myself from falling asleep!"
- Despair, worry, fear, e.g., "Something bad will happen next time!" or "Nobody can help me!"
- Other: _____________________

Insomnia Severity (ISI)

Beliefs and Attitudes About Sleep (MZS)

How important is sleep in your life?

- It is not important to me; I do not think about my sleep.
- I only think about my sleep occasionally.
- Sleep has a normal level of importance, like it does for most other people.
- I think about my sleep more often than other people do.
- I organize my life around not jeopardizing my sleep.

Emotional Well-Being (HADS)

Thank you very much for your participation!

After evaluating this online survey, we will conduct a follow-up study.
May we contact you for this purpose?
If yes, please provide your email address below or contact us at:
tanja.gruenberger@stud.plus.ac.at
Your email address will be used exclusively to contact you regarding this follow-up study. It will not be shared with third parties.

Thank you very much!
